# CITRIC: cold-inducible translational readthrough in the chloroplast of *Chlamydomonas reinhardtii* using a novel temperature-sensitive transfer RNA

**DOI:** 10.1186/s12934-018-1033-5

**Published:** 2018-11-24

**Authors:** Rosanna Young, Saul Purton

**Affiliations:** 1 Algal Research Group, Institute of Structural and Molecular Biology, University College London, Gower Street, London, WC1E 6BT UK; 2 Present Address: Department of Medicine, Sir Alexander Fleming Building, Imperial College London, South Kensington Campus, London, SW7 2AZ UK

**Keywords:** Microalgae, Chloroplast, Industrial biotechnology, Inducible expression, tRNA, Premature termination codon, Opal suppression

## Abstract

**Background:**

The chloroplast of eukaryotic microalgae such as *Chlamydomonas reinhardtii* is a potential platform for metabolic engineering and the production of recombinant proteins. In industrial biotechnology, inducible expression is often used so that the translation or function of the heterologous protein does not interfere with biomass accumulation during the growth stage. However, the existing systems used in bacterial or fungal platforms do not transfer well to the microalgal chloroplast. We sought to develop a simple inducible expression system for the microalgal chloroplast, exploiting an unused stop codon (TGA) in the plastid genome. We have previously shown that this codon can be translated as tryptophan when we introduce into the chloroplast genome a *trnW*_*UCA*_ gene encoding a plastidial transfer RNA with a modified anticodon sequence, UCA.

**Results:**

A mutated version of our *trnW*_*UCA*_ gene was developed that encodes a temperature-sensitive variant of the tRNA. This allows transgenes that have been modified to contain one or more internal TGA codons to be translated differentially according to the culture temperature, with a gradient of recombinant protein accumulation from 35 °C (low/off) to 15 °C (high). We have named this the CITRIC system, an acronym for cold-inducible translational readthrough in chloroplasts. The exact induction behaviour can be tailored by altering the number of TGA codons within the transgene.

**Conclusions:**

CITRIC adds to the suite of genetic engineering tools available for the microalgal chloroplast, allowing a greater degree of control over the timing of heterologous protein expression. It could also be used as a heat-repressible system for studying the function of essential native genes in the chloroplast. The genetic components of CITRIC are entirely plastid-based, so no engineering of the nuclear genome is required.

**Electronic supplementary material:**

The online version of this article (10.1186/s12934-018-1033-5) contains supplementary material, which is available to authorized users.

## Background

The green alga *Chlamydomonas reinhardtii* is regarded as a safe platform for the expression of recombinant proteins due to its lack of endotoxins and harmful viruses and its excellent performance in recent toxicology tests [1]. Cultivation is easily scaled up and can be carried out in volumes ranging from microplates [2] to photobioreactors and 100 L bags [3, 4]. Each cell contains a single chloroplast with an average of 83 copies of the circular 205 kb plastome [5]. In the chloroplast, transgenes can be integrated precisely into the genome by homologous recombination, are present in multiple copies per cell and are not subject to gene silencing effects. A wide range of proteins have been expressed in the *C. reinhardtii* chloroplast, as recently reviewed [4, 6–8]. These include complex multi-domain immunotoxins for cancer therapy [9], oral vaccine candidates for aquaculture [10], mosquitocidal proteins [11] and a cellulose-hydrolyzing enzyme [12]. This expression platform is capable of producing proteins with disulphide bonds or phosphorylation [13].

Inducible and tunable gene expression systems are frequently used to control the production of recombinant proteins and metabolic products in industrial biotechnology. Such tools are well-developed for both *Escherichia coli* and yeast (reviewed in [14, 15]) and allow production to be switched on after sufficient biomass has been accumulated, preventing the host transcriptional and translational machinery from being overwhelmed during the growth phase. In the case of metabolic engineering, such control also helps to avoid detrimental effects on the cell such as interference with host metabolic pathways or the accumulation of toxic products or intermediates.

Equivalent inducible and tunable systems are needed for plant and algal chloroplast platforms, and whilst some progress has been made for plants, development is complicated by the cross-talk between the nuclear and plastidial genetic systems.

There are two main mechanisms for the regulation of gene expression in the wild-type *C. reinhardtii* chloroplast, both controlled by nucleus-encoded proteins imported from the cytosol. Firstly, transcriptional regulation is thought to be mediated by a single sigma factor, RPOD, that responds to light levels to establish a circadian rhythm [16]. This contrasts with bacteria and higher plant chloroplasts, which generally use multiple sigma factors to control the transcription of subsets of genes in response to a variety of environmental or endogenous signals; in addition, higher plant chloroplasts have a second RNA polymerase. Secondly, *C. reinhardtii* regulates the maturation, stabilization (protection from nucleolytic degradation) or translational activation of plastidial transcripts [17]. Many of the nucleus-encoded factors that carry out these processes are highly target-specific; for example, NAC2 binds to the 5′ UTR of *psbD* mRNA only. Experimentally decreasing the plastome copy number or the overall transcription rate has little effect on the accumulation of chloroplast-encoded proteins [18, 19].

The development of inducible expression systems for the *C. reinhardtii* chloroplast has proved more challenging than in bacteria due to this reliance on the nucleus and predominantly post-transcriptional control. In one such system, *Nac2* is expressed using the copper-repressible cytochrome *c*_6_ promoter in the nucleus and the gene of interest is expressed using the *psbD* 5′ UTR in the chloroplast; this enables accumulation of the protein of interest upon removal of copper from the growth medium [20]. Whilst this setup is effective, it requires engineering of both the nuclear and plastidial genomes, and the 5′ UTR of the native *psbD* gene must be exchanged in order to maintain constitutive phototrophy. In addition, copper has toxic effects on *C. reinhardtii* [21, 22]. A variation on this system, using a *MetE* promoter and *Thi4* riboswitch for *Nac2*, allows control by vitamin B_12_ or thiamine rather than copper [23, 24]. The only other inducible system published for the *C. reinhardtii* chloroplast is based on the *E. coli* Lac repressor and requires IPTG [25]. A literature search suggests that this system has not been used in any subsequent projects.

Inducible systems developed for the tobacco chloroplast include a theophylline-dependent translational riboswitch [26, 27] and a nuclear-encoded ethanol-inducible T7 polymerase that enables expression of plastid genes under T7 promoters [28]; these tools have yet to be adapted for microalgae.

The use of temperature shifts to induce recombinant protein production is an attractive alternative to these chemical methods, particularly in a commercial context where induction needs to be cheap, simple and achievable at bioreactor scale. Natural isolates of *C. reinhardtii* have been recovered from across eastern North America and more recently from Japan [29, 30]; this species has therefore evolved to tolerate seasonal fluctuations in temperature, making this induction method potentially suitable. Vitova et al. [31] demonstrated that wild-type *C. reinhardtii* can undergo cell division across a temperature range of at least 15–37 °C, with the shortest cell cycles at 20–30 °C. Kremer et al. [32] found the highest growth rate to be at 30 °C, even when cells had been acclimated to 14 or 33 °C. A heat shock response, as measured by the transcript abundance of small heat shock proteins (chaperones) after 20 min, is induced at 36 °C but not 32 °C [33]. The *Hsp70A* heat shock promoter has been used for the inducible expression of nuclear transgenes at 40 °C [34].

In this paper we demonstrate a new inducible expression system for the *C. reinhardtii* chloroplast that is based on a method that we developed earlier for preventing transgene expression during cloning and for biocontainment. This method exploits the fact that the UGA stop codon is not used by any of the native genes in the *C. reinhardtii* plastome despite being capable of functioning as a terminator of translation [35], and comprises: (i) a copy of the native *trnW* (tryptophan tRNA) gene, termed *trnW*_*UCA*_ that has an altered anticodon sequence such that the tRNA (tRNA^Trp^-UCA) recognises UGA codons instead of UGG, and (ii) a gene of interest with one or more TGG-to-TGA codon alterations. Full-length translation of the gene of interest requires both components to be present, and operates only in *C. reinhardtii* since the chloroplast tRNA is not recognised in *E. coli* [35].

Here we developed a temperature-sensitive allele of *trnW*_*UCA*_ by investigating four different mutations predicted to affect the secondary structure of the tRNA. The first mutation was based on work by Marschalek et al. in 1990 [36], who developed a temperature-sensitive version of a *Dictylostelium discoideum* glutamic acid tRNA and tested it in *Saccharomyces cerevisiae*. The authors showed that their C to U transition in the acceptor stem led to a less stable secondary structure, so the pre-tRNA could be processed to a mature tRNA at 22 °C but not at 37 °C. We introduced the equivalent mutation into our plastidial *trnW*_*UCA*_ gene. The mutation in our second variant imitates that found in the tryptophan tRNA of a temperature-sensitive *E. coli* isolate [37, 38]. In *E. coli*, this mutation weakens the acceptor stem and makes it more susceptible to denaturation, especially at elevated temperatures [38]. Our third variant has altered base pairing in the dihydrouracil (D) loop and is based on work on tryptophan variants from *E. coli* [39]; it should be noted that in *E. coli*, altering this base (position 24) affected not just the tRNA stability but also the codon-anticodon recognition. Our fourth tRNA design is based purely on the principle that A-U base pairs have weaker hydrogen bonding than G-C pairs, so an alteration in the acceptor stem may destabilize the tRNA secondary structure sufficiently to disable function and/or processing at higher temperatures.

Of these four mutations, we found that the first conferred clear temperature sensitivity upon the synthetic tRNA in the *C. reinhardtii* chloroplast, enabling the development of a cold-inducible and tunable translation system. This eliminates the need for expensive chemical inducers and avoids the risk of contamination during the addition of such reagents.

## Results

### Variant 1 of tRNA^Trp^-UCA (tCI) allows temperature-dependent readthrough of internal UGA codons

**Fig. 1:**
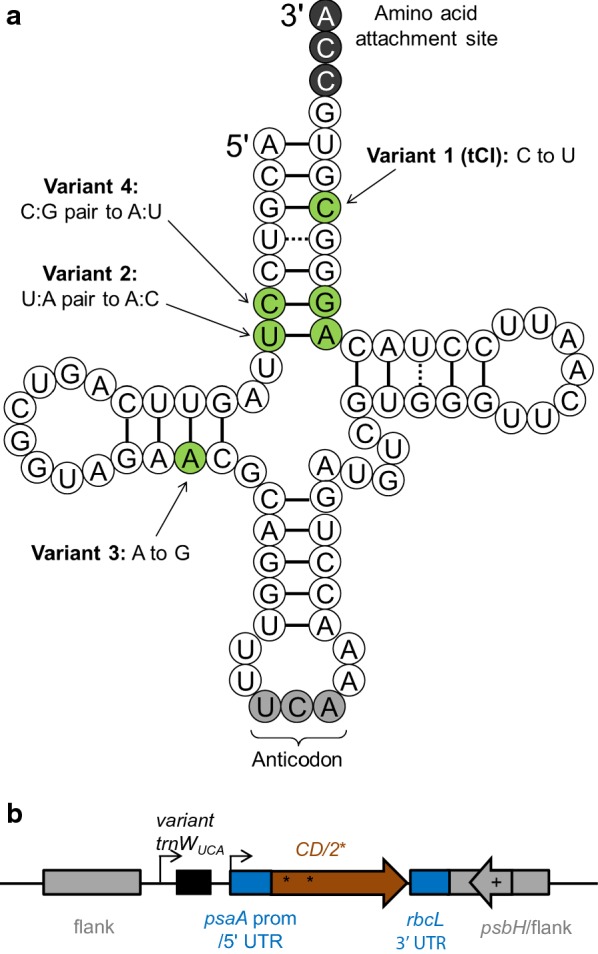
Designs for testing different tRNA^Trp^-UCA variants for temperature-sensitive behaviour. **a** Representation of the tRNA secondary structure with four variant versions annotated. Note the UCA anticodon (for UGA recognition) at the bottom of the structure. In chloroplasts, the 3′ CCA is added post-transcriptionally. Chemical modifications are not shown. **b** Construct design. Only the region that integrates into the chloroplast genome is shown; flanks are for homologous recombination into the genome. Each plasmid also contains an ampicillin resistance gene for selection during cloning in *E. coli*

We designed and tested four variants of the *trnW*_*UCA*_ gene in order to determine whether they would produce a temperature-dependent tRNA in the chloroplast that was still able to recognise internal UGA codons within the transcript of a transgene at a lower temperature, but not at a higher temperature. The variants each had one or two nucleotide changes from our original *trnW*_*UCA*_ gene (Fig. 1), with these changes designed to reflect known temperature-sensitive mutations in other organisms and/or a less negative free energy prediction to reduce stability of the RNA secondary structure (see “Background” section). The test transgene was *CD*, a cytosine deaminase gene of bacterial origin but codon-optimised for the *C. reinhardtii* chloroplast. We previously developed CrCD (encoded by *CD*) as a negative selectable marker [40] and chose to use it in the present study for its ease of detection using the C-terminal HA tag, clear phenotype of 5-fluorocytosine sensitivity, and non-toxicity to *E. coli* and *C. reinhardtii* on normal media.

**Fig. 2:**
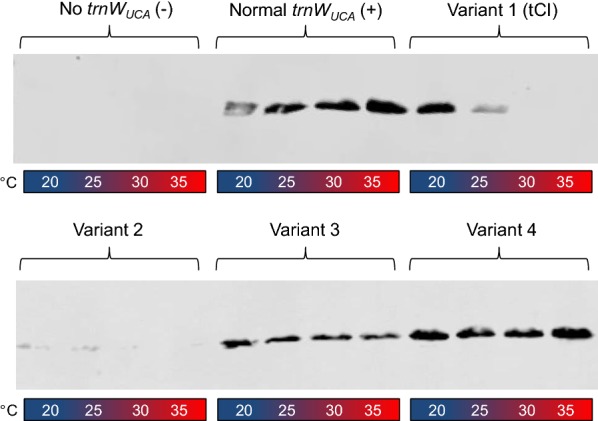
Analysis of the ability of the four tRNA variants to translate a test protein, CrCD, at different growth temperatures. Six *C. reinhardtii* cell lines, all containing the CD/2* gene but with different tRNA variants, were grown for 72 h at 20, 25, 30 or 35 °C. Crude extracts were equalised according to the culture optical density at 750 nm and subjected to SDS-PAGE and Western blotting using an anti-HA antibody to detect CrCD protein. See Additional file 1: Table S2 for culture conditions

**Fig. 3:**
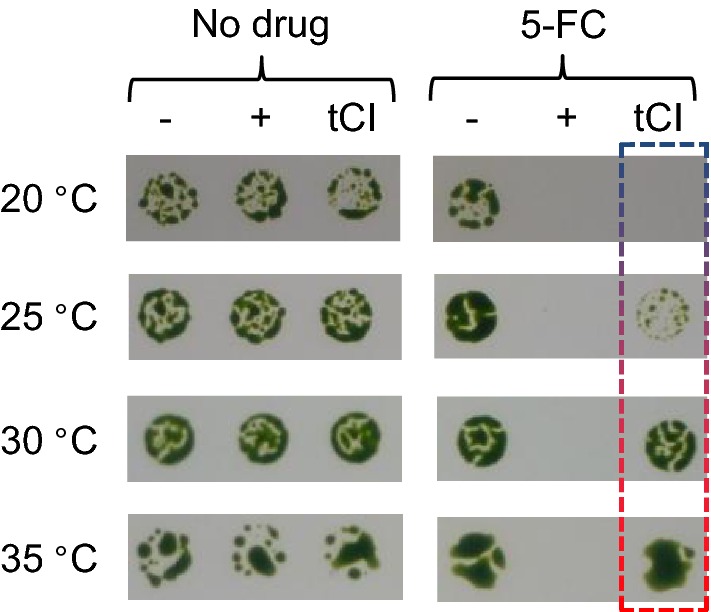
Growth test demonstrating that the introduction of *tCI* into the chloroplast genome allows the temperature-dependent translation of functional CrCD protein from the CD/2* gene. Liquid cultures grown at 35 °C were spotted onto TAP agar containing no drug (left panel) or 2 mg/ml 5-fluorocytosine (5-FC; right panel) then incubated at different temperatures for 10 days. Three *C. reinhardtii* cell lines were used, all containing the CD/2* gene. In the cell line with no additional tRNA gene (−), CD/2* is not translated so the cells grow on 5-FC. In the cell line with *trnW*_*UCA*_ encoding the constitutive version of the tRNA (+), CD/2* is translated at all growth temperatures and prevents growth on 5-FC. In the cell line with *tCI*, CrCD accumulation and subsequent growth inhibition depend on the temperature

We generated *C. reinhardtii* cell lines that contained a *CD* gene with two internal TGG-to-TGA mutations (CD/2*) and a *trnW*_*UCA*_ variant gene, both integrated downstream of *psbH* in the chloroplast genome (Fig. 1b). Full length CrCD protein (including the C-terminal HA tag) will only be produced when the tRNA variant allows translational readthrough of the internal UGA codons in the CD/2* transcript; we tested this by western blot analysis using anti-HA antibodies. The cell lines were grown at 20, 25, 30 and 35 °C for 72 h and the final level of CrCD protein was visualised by immunoblotting of whole cell extracts (Fig. 2). Cell lines containing *trnW*_*UCA*_ variant genes 2, 3 or 4 did not show marked variation in CrCD levels across the temperature range, so were not studied further. However, tRNA^Trp^-UCA variant 1, which harbours a C-to-U mutation in the acceptor stem, was found to display the desired temperature-dependent behaviour: more CrCD protein clearly accumulated at the lower temperatures (Fig. 2). Growth tests on media with or without 5-fluorocytosine, which CrCD converts to toxic 5-fluorouracil, confirmed this behaviour and demonstrated that the CrCD enzyme produced using tRNA^Trp^-UCA variant 1 was active (Fig. 3 and Additional file 1: Figure S1). The variant 1 gene was renamed ‘*tCI*’ and its corresponding tRNA ‘tCI’ to reflect its cold-inducible behaviour. Growth experiments show that there is no apparent deleterious effect from expressing *tCI* in *C. reinhardtii* (Additional file 1: Figure S2).

### Expression levels can be tailored by varying both induction temperature and the number of internal UGA codons

**Fig. 4:**
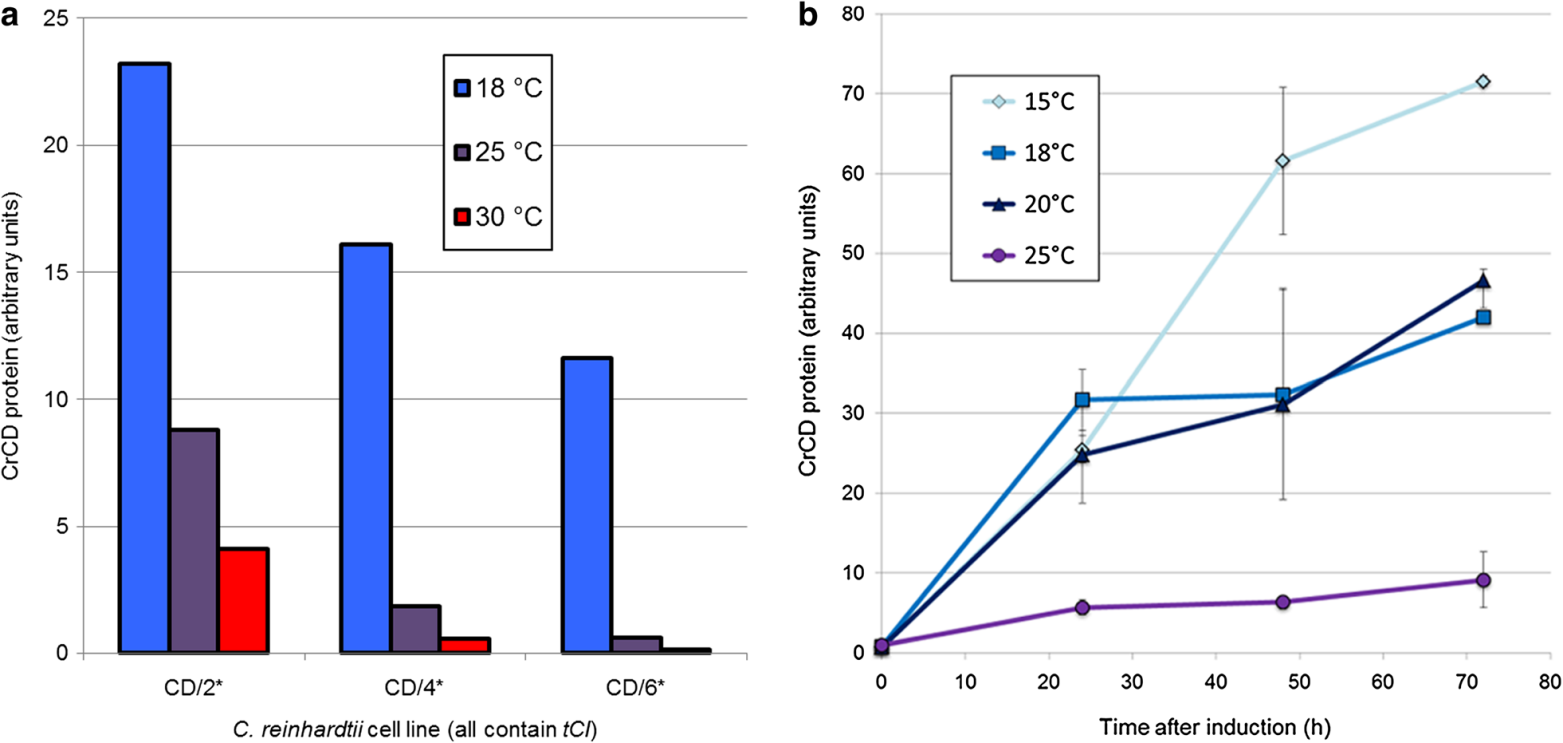
Investigation of CrCD protein accumulation in *C. reinhardtii tCI* cell lines upon varying the number of internal TGA codons and/or induction temperature. **a** Cell lines with 2, 4 or 6 internal TGA codons in the *CD* gene were pre-cultured at 30 °C then shifted to 18, 25 or 30 °C for induction. CrCD protein accumulation was measured by anti-HA immunoblot after 72 h. Equal culture volumes were used for each immunoblot sample, then anti-HA band intensity values were divided by the optical density (750 nm) of that culture to plot relative ‘per cell’ CrCD. Each bar shows the mean value for two cultures. **b** The CD/4* cell line was grown in four identical flask cultures at 35 °C to a high cell density (OD_750_ = 2.1) then induced at 15, 18, 20 or 25 °C using Algem photobioreactors. CrCD accumulation was followed for 72 h after induction. Samples were equalised according to optical density at 750 nm and analysed by SDS-PAGE and anti-HA immunoblotting. Error bars (on all data points) show ± SD for two samples taken from the same flask at each timepoint

To investigate whether the number of UGA codons within an mRNA affected its translational efficiency using tCI, we mutated the CD/2* plasmid that contained *tCI* to create versions with four or six TGG-to-TGA codon changes within the *CD* gene rather than two (see Sequences 3 and 4 in Additional File 1). These were used to generate *C. reinhardtii* transformants for immunoblotting experiments (Fig. 4a). Increasing the number of internal UGA codons led to a tighter OFF state at 30 °C, with good repression of test protein accumulation at high growth temperatures. This would be suitable for recombinant proteins that are highly toxic to the host cell even at low concentrations. However, the maximum CrCD protein yield upon induction was lower, suggesting that the amount of active tCI is rate-limiting when four or six UGA codons are present. The yield for a particular recombinant protein could therefore be tailored by a combination of genetic element choice (promoter and UTRs), UGA number and induction temperature.

### Maximum protein accumulation is achieved at 15 °C

To determine the best temperature for induction and protein accumulation, the CD/4* + *tCI* cell line of *C. reinhardtii* was grown to late exponential phase at 35 °C (‘growth phase’) then cooled to a range of temperatures (‘induction phase’). The level of CrCD protein accumulation over the next 72 h was tracked by immunoblotting. As shown in Fig. 4b, CrCD accumulated over the 72 h under all induction temperatures tested (15–25 °C), but reached the highest levels at 15 °C. A second experiment demonstrated that induction at an even lower temperature of 12 °C is still efficient but did not improve the CrCD yield further (Additional file 1: Figure S3; the CD/2* + *tCI* cell line was used in this case).

Growth curves following induction are shown in Additional file 1: Figure S4 for the first experiment, and show similar biomass yields reached after approximately 48 h as the cultures enter stationary phase. The maximum production of the CrCD protein per unit of biomass is therefore seen following incubation of the culture at 15 °C for at least 72 h, although the optimum timepoint for harvesting a protein of interest would depend on its stability at the induction temperature, as the protein level depends upon the rate of both translation and degradation.

### Inducible production of a *cis*-abienol synthase enzyme, TPS4

**Fig. 5:**
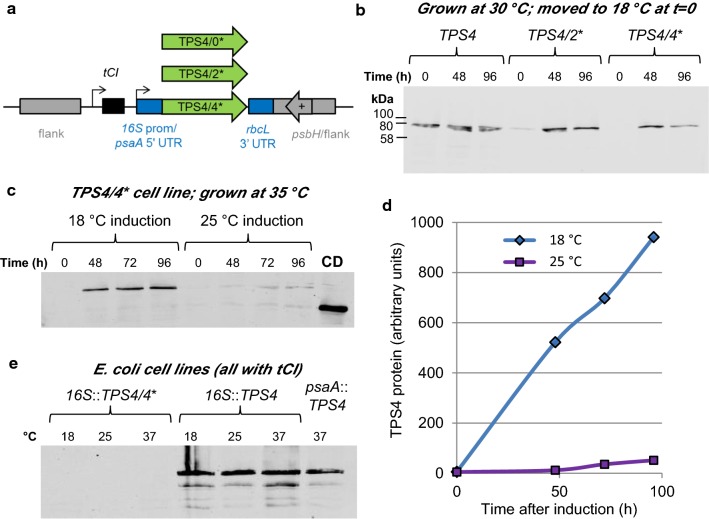
Cold-inducible synthesis of *cis*-abienol synthase, TPS4. **a** Design of chloroplast expression constructs. The *TPS4* genes include a C-terminal HA tag for detection. **b** Immunoblot using anti-HA antibodies to detect TPS4 protein in *C. reinhardtii tCI* cell lines induced at 18 °C. Samples were equalised according to the culture optical density at 750 nm before loading. The first three lanes show a constitutive *TPS4* control cell line with no internal TGA codons. **c** Anti-HA immunoblot to compare induction temperatures of 18 and 25 °C in *C. reinhardtii*. Samples were equalised according to the culture optical density at 750 nm before loading. A strain synthesising CrCD (right lane) is included as a negative control for the TPS4 band. **d** Quantification of the band intensities in part c, showing the TPS4 induction time-course at two temperatures. **e** Anti-HA immunoblot to detect TPS4 protein in *E. coli* DH5α containing various *TPS4* chloroplast expression plasmids. Total protein stain for this blot is shown in Additional file 1: Figure S6. The incubation temperature is given above each lane

Inducible systems can be useful in the field of metabolic engineering as they allow the accumulation of cell mass prior to switching on a potentially detrimental enzymatic pathway. For example, some terpenes have cytotoxic effects on the production host (reviewed in [41]). Constitutive expression of the *TPS4* gene, encoding a bifunctional *cis*-abienol synthase from *Abies balsamea* (Balsam Fir), in the *C. reinhardtii* chloroplast produces a slight but reproducible growth defect [42, 43]. This enzyme converts geranylgeranyl diphosphate to *cis*-abienol, which is a diterpene that can be used as a precursor for Ambrox fragrances [44]. By creating two or four TGG-to-TGA mutations in the codon-optimised *TPS4* open reading frame and inserting it alongside *tCI* into the *C. reinhardtii* chloroplast genome (Fig. 5a), we were able to induce TPS4 protein accumulation upon moving the cultures from 30 °C to 18 °C (Fig. 5b). Figure 5c, d show the effect of inducing TPS4 at 18 °C (high expression) versus 25 °C (low expression) for the *C. reinhardtii* cell line with four internal TGA codons in *TPS4*.

The *C. reinhardtii* TPS4 cell lines used for Fig. 5 were developed using our standard glass bead transformation procedure, which involves incubating the minimal media transformation plates at 25 °C for 2–4 weeks to allow the selection of phototrophic transformant colonies through restoration of *psbH* [45]. However, we found that homoplasmic transformants could also be obtained from plates incubated at 30 °C (Additional file 1: Figure S5). This could be useful for genes of interest where tighter repression is required during cell line production due to high levels of toxicity.

### Informational biocontainment: chloroplast promoters are functional in *E. coli*, but tCI is not

We previously demonstrated that when using *E. coli* as a cloning host, the *C. reinhardtii psaA* promoter and 5′ UTR element allow expression of transgenes in this bacterium [35, 45]. This is also true for the *C. reinhardtii* 16S rRNA promoter plus *psaA* 5′ UTR combination (Fig. 5e) and is a potential problem when cloning antibacterial or metabolic enzymes that might prove toxic to *E. coli*. However, the constitutive tRNA^Trp^-UCA does not function in *E. coli*, so a construct containing *trnW*_*UCA*_ plus a gene of interest with internal TGA codons will provide protection against transgene expression during cloning in *E. coli* [35]. Figure 5e shows that the same holds true for the inducible tRNA, tCI. As this tRNA only functions at low temperatures in *C. reinhardtii*, we allowed *E. coli* to grow to mid-log phase (3 h at 37 °C) then incubated the cultures at a range of temperatures for 18 h; no accumulation of the test protein (TPS4/4*) was observed using anti-HA immunoblotting (Fig. 5e and Additional file 1: Figure S6).

### Translational readthrough in the *C. reinhardtii* chloroplast is not inducible by drugs

Readthrough of premature termination codons (PTCs) is known to be triggered by certain chemicals, including some aminoglycosides, a phenomenon exploited in the search for treatments for PTC-associated genetic diseases such as cystic fibrosis [46, 47]. Low concentrations of erythromycin and chloramphenicol enhance promiscuous UGA readthrough in *E. coli* [48]. To investigate whether UGA readthrough could be drug-induced in the *C. reinhardtii* chloroplast, we incubated a cell line that contains CD/2* but no added tRNA genes in media containing various low concentrations of kanamycin, chloramphenicol, spectinomycin or erythromycin for 48 h. However, we did not observe a significant increase in the level of CrCD protein above the trace amount seen in the control in any of the conditions tested (Additional file 1: Figure S7). Using the *tCI* tRNA gene therefore remains the best way to allow inducible readthrough of transgenes containing TGA codons in the *C. reinhardtii* chloroplast.

### Plasmids available for the CITRIC system

**Fig. 6:**
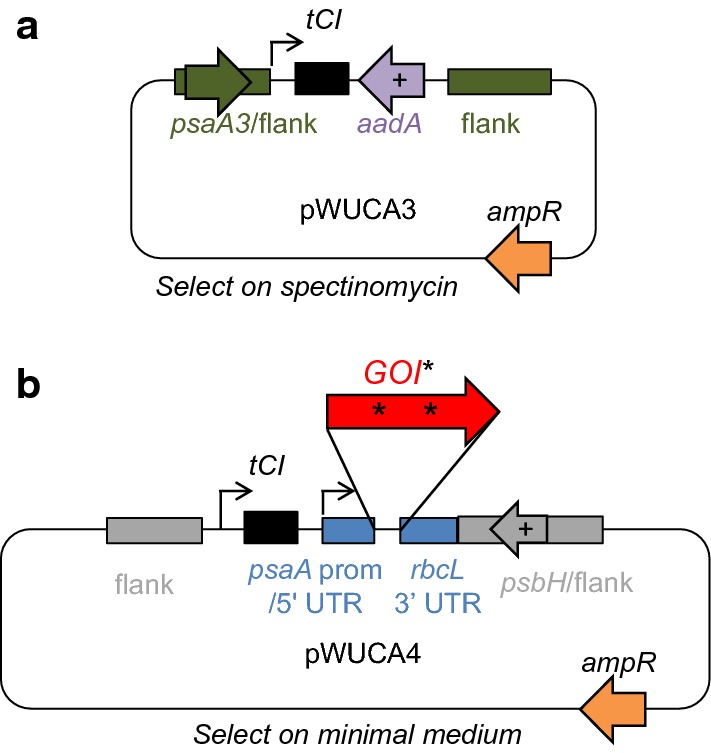
Plasmids available to introduce the CITRIC system into the *C. reinhardtii* chloroplast. The induction system needs only the *tCI* tRNA gene, plus a gene of interest containing internal TGA codons. The ampicillin resistance gene (*ampR*) is for selection in *E. coli* and is outside the region that integrates into the chloroplast genome. **a** pWUCA3, **b** pWUCA4

Two plasmids are available for researchers wishing to use the CITRIC system to induce the accumulation of proteins of interest in the *C. reinhardtii* chloroplast (Fig. 6). Plasmid pWUCA3 carries the cold-inducible *trnW*_*UCA*_ gene (*tCI*) plus an *aadA* cassette for selection using spectinomycin and streptomycin resistance. This integrates into the chloroplast genome within an intergenic region downstream of *psaA* exon 3. This plasmid can be used in any *C. reinhardtii* cell line; the gene of interest containing internal TGA codons would be introduced separately. Plasmid pWUCA4 contains *tCI*, an intact copy of *psbH* for phototrophic selection, and an empty expression cassette into which the gene of interest can be introduced using restriction enzymes SapI and SphI. This plasmid is designed for the transformation of a *psbH* deletion mutant such as *C. reinhardtii* TN72 and uses the intergenic region downstream of *psbH* as the integration site [45]. DNA sequences of both plasmids are given in Additional file 1, and the workflow for pWUCA4 is shown in Box 1.

## Box 1. Workflow for the CITRIC system: inducible transgene expression

1. Order the gene of interest, codon-optimised for the *C. reinhardtii* chloroplast and with 2–6 mutated Trp codons (TGG to TGA), preferably near the N-terminus to reduce translational burden. Include a C-terminal epitope tag if required for protein detection or purification. Include a SapI site at 5′ end for precise fusion to 5′ UTR, and an SphI site at 3′ end.
2. Ligate into empty expression cassette in pWUCA4 using SapI and SphI. Clone in *E. coli*.
3. Use the resulting plasmid to transform *C. reinhardtii* TN72. Select on minimal media at 25 °C (or 30 °C if protein considered highly toxic) for restoration of phototrophy due to *psbH* gene in pWUCA4. Colonies appear in 2–4 weeks.
4. Confirm transgene integration and homoplasmy by PCR.
5. Identify optimal temperatures for the growth and induction phases. This may depend on the transgene and number of TGA codons used, but is likely to be 30–35 °C for the growth (repression) phase and 15–20 °C for full induction.

N.B. The system can be adapted for other plasmids and *C. reinhardtii* cell lines. Only the modified gene of interest, the *tCI* gene and a selectable marker are required.

## Discussion

Four variants of tRNA^Trp^-UCA were tested for their function at different temperatures (Figs. 1 and 2). Structural stability and correct folding of the tRNA are prerequisites for functionality. In silico secondary structure predictions using RNAfold software [49] shed some light on the observed results. First, the free energy prediction of variant 1 (tCI) is the least negative, indicating that this is the least stable of all the variants tested (Additional file 1: Figure S8). This is likely to contribute to its loss of function at elevated temperatures, either directly through unfolding or through an inability to fold correctly for pre-tRNA processing, making it useful for the CITRIC system. Second, whilst the structure predicted to have the minimum free energy was a classic tRNA cloverleaf shape for all combinations of tRNA and temperature shown in Additional file 1: Figure S8, the acceptor stem in variant 2 consisted of only six base pairings instead of seven. This is due to the mutation creating a non Watson-Crick match (Fig. 1a); an equivalent mutation creates a thermolabile tryptophan tRNA in *E. coli* [37], but appears to almost ablate function in *C. reinhardtii* (Fig. 2).

CITRIC could be used for the production of proteins (vaccines, antibodies, hormones, etc.) or for metabolic engineering. If the product of interest is the induced protein itself, this can be harvested after the cold induction phase. Indeed, temperatures of 6–25 °C are used in chemically-induced *E. coli* protein expression systems to aid protein stability and/or solubility [15]. However, if the induced protein is an enzyme for metabolic engineering within the chloroplast, the optimum temperatures for substrate availability, enzyme activity and product stability must be taken into account. For some reactions, sufficient product may be obtained during the cold induction phase, whereas for others a warmer incubation phase may be required following induction. Enzymes originally found in psychrophiles (cold-adapted organisms) are more likely to have efficient low-temperature activity and could be useful in this context [50, 51].

The generation of cell lines in which a certain essential endogenous gene is under temperature regulation can be valuable for assigning function [52], as constitutive knockout of such genes is not possible. The CITRIC system could be used to study chloroplast genes of unknown function such as *orf1995*, a large open reading frame for which there is expression evidence at both the transcript and protein level [5]. Such experiments will be easier now that a more accurate *C. reinhardtii* chloroplast genome sequence is available [5].

Another potential use for this system is the generation of markerless chloroplast transformants in any cell line. A constitutive selectable marker together with an inducible counter-selectable marker would be flanked by direct repeats, and selection carried out at the uninduced temperature. Once homoplasmy is established, translation of the counter-selectable marker would be cold-induced to select for homologous recombination between the repeats and loss of both marker genes. A similar strategy has been employed in *E. coli* using an IPTG-inducible *ccdB* toxin gene [53]. Possible alternatives to *ccdB* include the MazF endoribonuclease family [54–56] or toxin genes from toxin-antitoxin systems [57].

The native *C. reinhardtii* chloroplast tRNA^Trp^ encoded by *trnW* translates UGG codons as tryptophan. The tRNA^Trp^-UCA version of it that we developed previously has a mutated anticodon so that it recognises UGA codons (Fig. 1a); we inferred that this tRNA still adds tryptophan, as it was able to rescue a TGG-to-TGA mutation that affected an essential tryptophan residue in PsaA [35]. The tCI tRNA used in the CITRIC system has one further mutation, namely a C-to-U transition in the acceptor stem at position 70 (Fig. 1a). We are confident that the majority of residues incorporated at CITRIC TGA sites are tryptophan, as purified CrCD protein from a C. reinhardtii CD/6* tCI strain (where 6 of the 7 TGG codons in *CD* are replaced with TGA) gives an equivalent strength signal with a tryptophan-specific binding compound when compared to control CrCD protein (Additional file 1: Figure S9). Nevertheless, the recognition of tRNAs by their cognate aminoacyl tRNA synthetases (i.e. amino acid specificity) can be complex [58], so it is recommended that the amino acid sequences of proteins expressed using the CITRIC system are verified if incorporation of 100% tryptophan at UGA codon positions is required.

It should be noted that internal UGA readthrough has been observed by others in *E. coli* when a strong inducible bacterial promoter, single UGA codon and deliberately readthrough-enhancing RNA context is used (e.g. placing a GAC codon two positions upstream) [59]. The use of chloroplast promoters and inclusion of more than one internal UGA codon in the gene of interest will help to prevent readthrough in *E. coli* when using CITRIC, but this should be confirmed experimentally for each gene of interest if a lack of readthrough is being relied upon for biocontainment.

The possibilities and limitations of adapting the artificial tRNA system for other microalgal or higher plant chloroplasts was discussed previously [35]. In brief, this depends on the availability of free or rarely-used codons in the chloroplast genome, in addition to the existence of plastid transformation technology for that species. The same criteria apply for the CITRIC system.

## Conclusions

We have developed a cold-inducible translation system for the *C. reinhardtii* chloroplast using a tRNA that can read through internal UGA codons at low temperatures only. This increases the scope of the organism as a platform for heterologous protein production, metabolic engineering and the study of essential genes. We hope that it will prove a useful addition to the genetic engineering toolbox for microalgae.

## Methods

### Algal strains, culture conditions and transformation

All *C. reinhardtii* cell lines used in this research were made from the parental line TN72 [40], which is a *cw15* (i.e. cell wall deficient) mutant with an *aadA* spectinomycin resistance cassette replacing part of *psbH* in the chloroplast genome. TN72 is available from the Chlamydomonas Resource Center (http://www.chlamycollection.org) as strain CC-5168. Transformation of TN72 was performed using the glass bead vortexing method [40] and a plasmid containing an intact copy of *psbH* in addition to the genes of interest, resulting in markerless phototrophic transformants whose homoplasmicity was confirmed by PCR as described previously [35]. DNA sequencing (Source BioScience, Nottingham, UK) was used to confirm that the introduced genetic elements were intact. *C. reinhardtii* was grown in Tris-acetate phosphate (TAP) medium [60], with 2% agar (Fisher Bioreagents, New Hampshire, USA) added for solid plates. For selection of phototrophic transformants, high-salt minimal (HSM) medium with 2% agar was used [60]. Liquid cultures were grown in glass flasks or in clear plastic 12 well tissue culture plates (VWR, Pennsylvania, USA). For some experiments (see Additional file 1: Table S2), two parallel Algem photobioreactors were used (Algenuity, Stewartby, UK).

### Plasmid construction

To test *trnW*_*UCA*_ variant genes 1 to 4 (see Sequence 2 in Additional file 1), certain single or double point mutations were introduced into the *trnW*_*UCA*_ region of plasmid pWUCA2-CD** [35] by PCR amplification using the primers in Additional file 1: Table S1 followed by Gibson assembly [61] and cloning using chemically competent *E. coli* DH5α [62]. Plasmid pWUCA4 was prepared similarly from pWUCA2 [35], introducing a single point mutation to change *trnW*_*UCA*_ to *tCI*. The same applies to pWUCA3, created from pWUCA1 [35]. To create the *TPS4* plasmids in Fig. 5a, a *TPS4 cis*-abienol synthase gene with a C-terminal HA tag and codon optimisation for the *C. reinhardtii* chloroplast was obtained from Julie Zedler (University of Kent, UK) and cloned between the SapI and SphI sites in pWUCA4. The *psaA* exon 1 promoter was replaced with a 16S rRNA promoter amplified from *C. reinhardtii* using Gibson assembly; see Sequence 7 in Additional file 1. The *psaA* 5′ UTR was retained. The *C. reinhardtii* 16S rRNA promoter has been shown by others to give high expression levels for other transgenes [12, 63].

Internal stop codons (TGG-to-TGA mutations) were introduced into *CD* and *TPS4* at the positions indicated in the supplementary sequence data (Additional file 1): the relevant plasmid was amplified using primers spanning each mutation site in the format N_10_(G to A)N_13-20_ for the forward primer and N_10_(C to T)N_13-20_ for the reverse primer, and the PCR products were reassembled using Gibson assembly. All PCR amplification was carried out using Phusion High-Fidelity DNA Polymerase, and plasmids were prepared for algal transformation using a GeneJET Plasmid Midiprep Kit (both ThermoFisher, Waltham, USA).

### 5-fluorocytosine sensitivity test

For the growth test in Fig. 3, 20 ml TAP cultures of the three *C. reinhardtii* cell lines were grown at 35 °C for 48 h. Cultures were then diluted to OD_750_ = 0.2 and 5 µl was spotted onto TAP + 2% agar plates that contained 0 or 2 mg/ml 5-fluorocytosine, which was prepared as a 5X stock in TAP medium. Plates were incubated under 25 µE/m^2^/s light at 20, 25, 30 or 35 °C and photographed after 10 days.

### *C. reinhardtii* immunoblots

Growth conditions for each induction experiment are given in Additional file 1: Table S2. Cells were harvested by centrifugation (4000*g*, 5 min) and resuspended in 0.8 M Tris/HCl pH 8.3, 0.2 M sorbitol and 1% β-mercaptoethanol to equal cell densities, as measured by light scattering of the cell culture at 750 nm. The exceptions to this were Fig. 4a and Additional file 1: Figure S7, where equal culture volumes were used instead (see figure legends). SDS-PAGE, blotting onto nitrocellulose membranes and antibody probing were performed as described previously [35], i.e. all membranes were probed with 1:5000 rabbit αHA primary antibody (Sigma-Aldrich, Missouri, USA) then 1:25,000 goat α-rabbit Dylight 800 secondary antibody (ThermoFisher). HA-tagged CrCD and TPS4 proteins were then detected using an Odyssey CLx imaging system at 800 nm and analysed in Image Studio (both LI-COR, Nebraska, USA).

### *E. coli* immunoblot

*Escherichia coli* DH5α cultures containing each of the three TPS4 plasmids were grown in 5 ml LB with 100 µg/ml ampicillin for 3 h at 37 °C. Optical densities were then adjusted to 0.75 at 600 nm, and 1 ml cultures were transferred to 18, 25 or 37 °C incubators, shaking at 200 rpm for 18 h. The final optical densities at 600 nm were in the range 2.5–6.0 depending on temperature. Cultures were pelleted and resuspended in sample buffer to equal optical densities following the Mini-PROTEAN Tetra Cell manual (Bio-Rad, California, USA). 200 µl samples were boiled for 2 min and centrifuged for 2 min at 13,000*g*, then 10 µl of the supernatant was separated on a 15% acrylamide SDS-PAGE gel (100 V, 150 min). The gel was blotted onto a Hybond ECL nitrocellulose membrane (GE Healthcare, Illinois, USA) using a Trans-blot semi-dry transfer cell (Bio-Rad) run at 20 V for 1 h. The membrane was stained using REVERT Total Protein Stain (LI-COR) and imaged at 700 nm to confirm equal loading and transfer. After blocking overnight at 4 °C in TBS-T + 5% milk, the membrane was probed and imaged as above except that the primary antibody concentration was 1:25,000.

## Additional file

**Additional file 1: Figure S1.** Testing tRNA^Trp^-UCA variants for temperature-dependent behaviour. **Figure S2.** Growth curve of *C. reinhardtii* cell lines, showing that the temperature-sensitive tRNA (tCI) does not cause a growth defect. **Figure S3.** Induction of CrCD protein in the C. reinhardtii CD/2* + *tCI* cell line at low temperatures. **Figure S4.** Growth curves for C. reinhardtii CD/4* + *tCI* following induction at 15–25 °C. **Figure S5.** PCR confirming homoplasmic transgene integration into *C. reinhardtii* TN72 using transformation plates incubated at 30 °C. **Figure S6.** REVERT total protein staining of *E. coli* western blot membrane in Fig. 5e, confirming equal loading and blotting across lanes. **Figure S7.** Investigation of readthrough effect of four drugs as an alternative method of induction. **Figure S8.** Minimum free energy calculations for each tRNA variant at 15–35 °C. **Figure S9.** Detection of tryptophan in purified CrCD protein from *C. reinhardtii CD* (positive control) and CD/6* + *tCI* cell lines. **Table S1.** Primers used to alter *trnW*_*UCA*_ to make variants 1 to 4. **Table S2.** Conditions for *C. reinhardtii* induction experiments. **Sequences 1 to 9.** Includes DNA sequences for tRNAs, genes *CD* and *TPS4*, 16S promoter and plasmids pWUCA3 and pWUCA4.

## Abbreviations

5-FC: 5-fluorocytosine
CITRIC: cold-inducible translational readthrough in chloroplasts
CrCD: cytosine deaminase protein
PTC: premature termination codon
TAP: tris-acetate-phosphate medium
tCI: tRNA^Trp^-UCA variant 1 (cold-inducible)
*tCI*: *trnW*_*UCA*_ *variant 1* gene
