# Supplementary material for: CITRIC: cold-inducible translational readthrough in the chloroplast of Chlamydomonas reinhardtii using a novel temperature-sensitive transfer RNA
Source: Microb Cell Fact. 2018 Nov 24;17:186. doi: 10.1186/s12934-018-1033-5 (PMC6260665; doi:10.1186/s12934-018-1033-5)
Supplement: Supplementary file 1 — Additional file 1: Figure S1. Testing tRNATrp-UCA variants for temperature-dependent behaviour. Figure S2. Growth curve of C. reinhardtii cell lines, showing that the temperature-sensitive tRNA (tCI) does not cause a growth defect. Figure S3. Induction of CrCD protein in the C. reinhardtii CD/2* + tCI cell line at low temperatures. Figure S4. Growth curves for C. reinhardtii CD/4* + tCI following induction at 15–25 °C. Figure S5. PCR confirming homoplasmic transgene integration into C. reinhardtii TN72 using transformation plates incubated at 30 °C. Figure S6. REVERT total protein staining of E. coli western blot membrane in Fig. 5e, confirming equal loading and blotting across lanes. Figure S7. Investigation of readthrough effect of four drugs as an alternative method of induction. Figure S8. Minimum free energy calculations for each tRNA variant at 15–35 °C. Figure S9. Detection of tryptophan in purified CrCD protein from C. reinhardtii CD (positive control) and CD/6* + tCI cell lines. Table S1. Primers used to alter trnWUCA to make variants 1 to 4. Table S2. Conditions for C. reinhardtii induction experiments. Sequences 1 to 9. Includes DNA sequences for tRNAs, genes CD and TPS4, 16S promoter and plasmids pWUCA3 and pWUCA4. [file 12934_2018_1033_MOESM1_ESM.docx]

***Additional File 1***

**CITRIC: Cold-inducible translational readthrough in the chloroplast of *Chlamydomonas reinhardtii* using a novel temperature-sensitive transfer RNA.**

Rosanna Young and Saul Purton

**SUPPLEMENTARY FIGURES**

**Figure S1. Testing tRNA^Trp^-UCA variants for temperature-dependent behaviour.** *C. reinhardtii* growth assay in liquid media containing 0 or 2 mg/ml 5-fluorocytosine (5-FC) to establish effectiveness of different tRNA^Trp^-UCA variants in translating CrCD protein at 20-30 °C. 2.5ml TAP cultures at a starting OD_750_ of 0.2 were grown in 12-well plates under 25 µE light for 48 h. The pre-culture temperature was 35 °C. All six cell lines contain the *CD/2** gene; the CrCD (cytosine deaminase) enzyme converts 5-FC to toxic 5-fluorouracil, so low survival equates to high CrCD expression. Note that the '*trnW_UCA_* normal' cell line will already be expressing CrCD at time zero, so the killing effect will be enhanced in this cell line and in any other constitutively expressing lines.

**Figure S2.** **Growth curve of *C. reinhardtii* cell lines, showing that the temperature-sensitive tRNA (tCI) does not cause a growth defect.** 200 ml TAP cultures were grown in the light at 20 °C. Note that *trnW_UCA_* has previously been demonstrated not to cause a growth defect (Young & Purton 2016, supplementary data).

**Figure S3.** **Induction of CrCD protein in the *C. reinhardtii* *CD/2** + *tCI* cell line at low temperatures.** A TAP culture was diluted to OD_750_ = 0.1 and split between flasks to make three identical 450 ml cultures. These were grown for 72 h at 35 °C, 120 rpm shaking, 200 µE/m^2^/s light in an Algem photobioreactor. At this point they had reached OD_750_ = 2.5-2.7. The temperatures were then altered to 12, 15 or 35 °C for the induction phase. Samples were equalised according to optical density at 750 nm and analysed by SDS-PAGE and anti-HA immunoblotting. Two samples were taken per flask at each timepoint; error bars show ±SD for these. If necessary, repression at 35 °C can be tightened by using more than two internal TGA codons.

**Figure S4. Growth curves for *C. reinhardtii* *CD/4** + *tCI* following induction at 15-25 °C.** Four identical 450 ml cultures were grown at 35 °C to an OD_750_ of 2.1-2.3, then the temperature was lowered to induce tCI activity. Error bars on all data points show +/- SD for two samples taken from each flask at each timepoint. These cultures were used for the immunoblot quantified in Fig. 4b. See Table S2 for culture conditions.


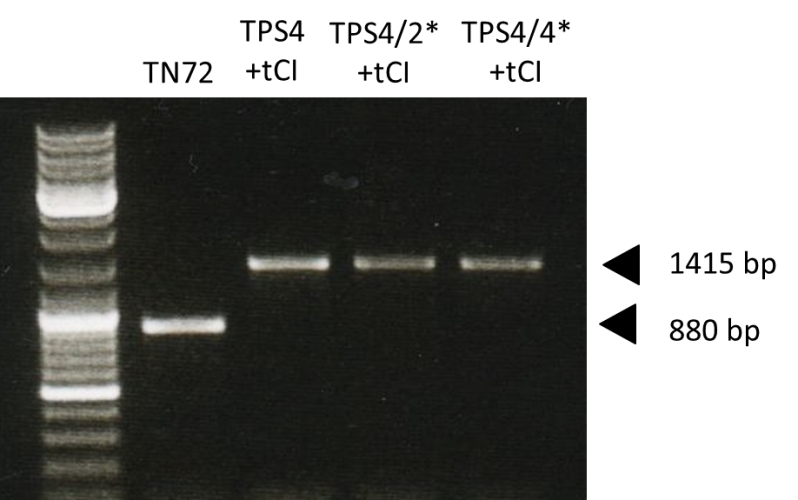


**Figure S5. PCR confirming homoplasmic transgene integration into *C. reinhardtii* TN72 using transformation plates incubated at 30 °C.** One colony was tested for each of three plasmids. These PCRs confirm that selection of transformants on minimal medium (HSM) for the restoration of the full-length *psbH* gene can be carried out at 30 °C as an alternative to the standard temperature of 25 °C. Three primers were used in each reaction; primer sequences are given in Young & Purton (2016), Table S2, last row. The expected product size for the parental strain, TN72, is 880 bp and that for homoplasmic integration events is 1415 bp; a heteroplasmic cell line would yield both bands.


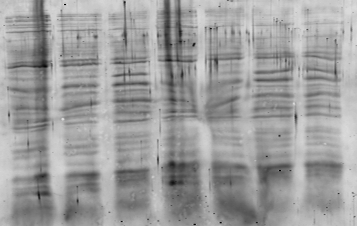


**Figure S6. REVERT total protein staining of *E. coli* western blot membrane in Figure 5e, confirming equal loading and blotting across lanes.**

**(a)**


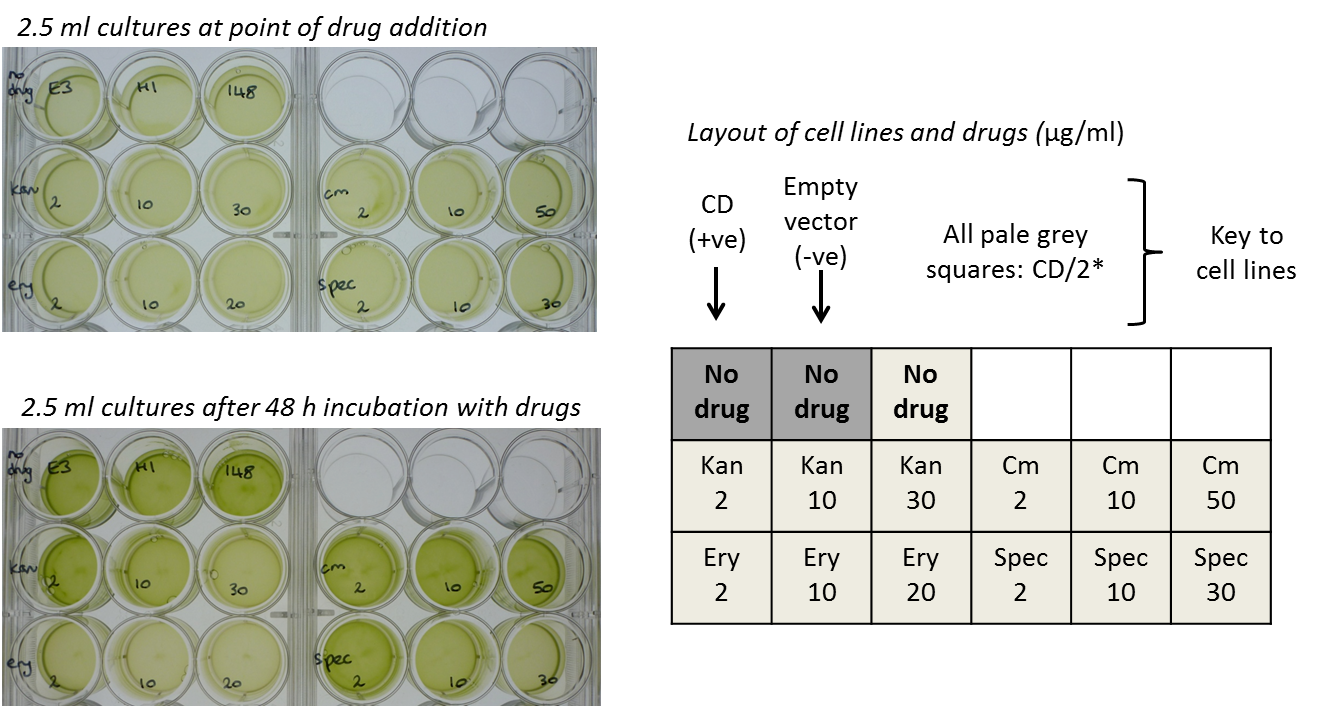


**(b)**


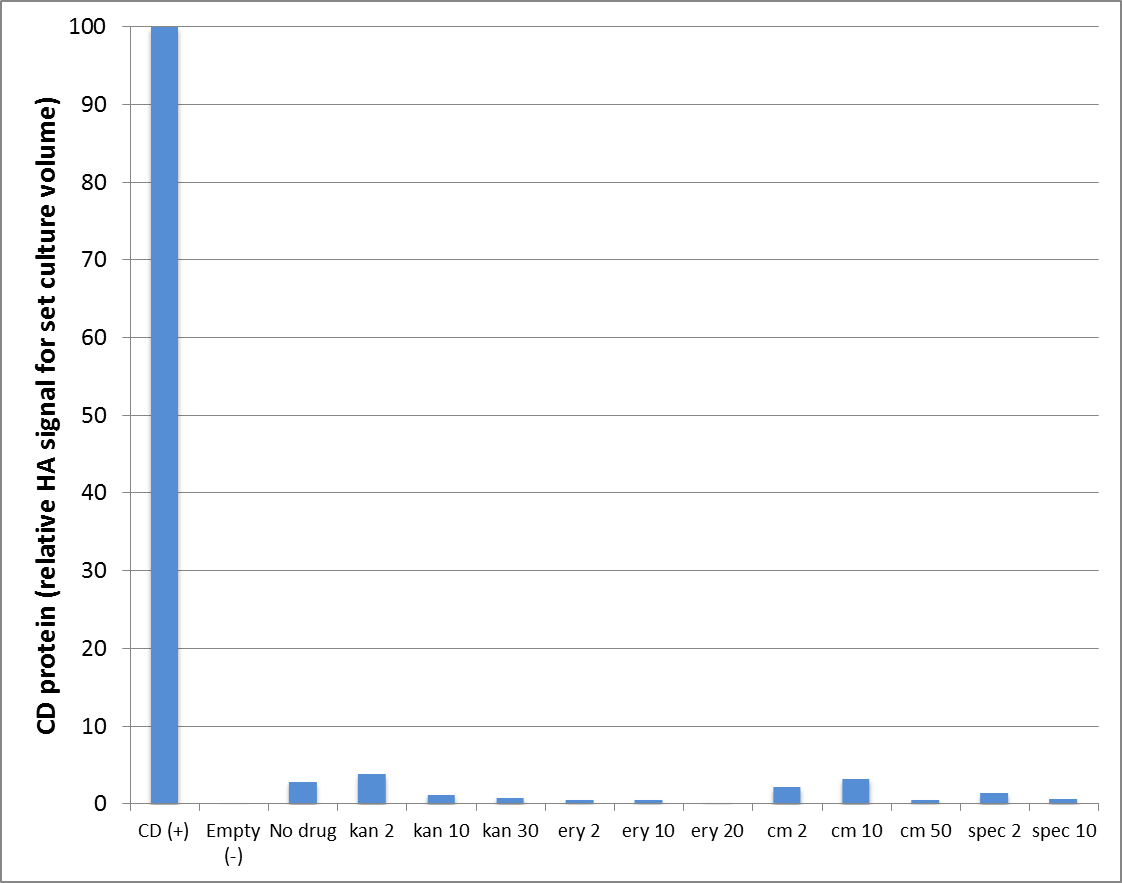


**Figure S7. Investigation of readthrough effect of four drugs as an alternative method of induction.**

*C. reinhardtii* cultures (2.5 ml) were incubated with low concentrations of kanamycin (kan), erythromycin (ery), chloramphenicol (cm) and spectinomycin (spec) for 48 h to investigate whether they allowed promiscuous readthrough of the two internal TGA codons in *CD/2** in the absence of the *tCI* gene. The starting density for each well was OD_750_ = 1.0; 12-well plates were incubated at 25 °C under medium light with no shaking. **a** Visualisation of culture growth and layout of experiment. Some of the drugs clearly affected cell growth at some concentrations. **b** Quantification of immunoblot using anti-HA antibody to detect CrCD protein. The signal from equal culture volumes were compared to that of the constitutive *psaA::CD* cell line (+).

**
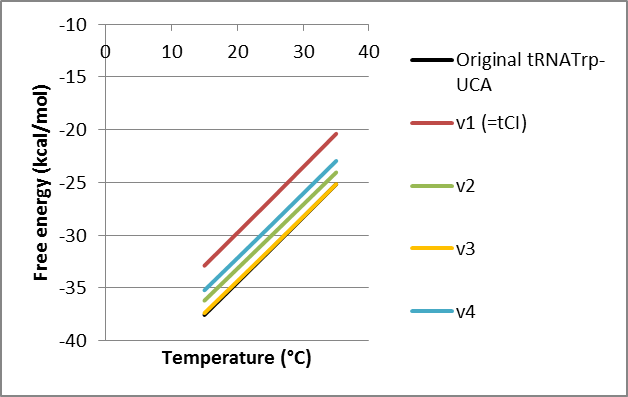
**

**Figure S8. Minimum free energy calculations for each tRNA variant at 15-35 °C.** Calculated using the RNAfold web server at <http://rna.tbi.univie.ac.at/> . These calculations do not take into account any nucleoside chemical modifications that may be present in the tRNAs.

**
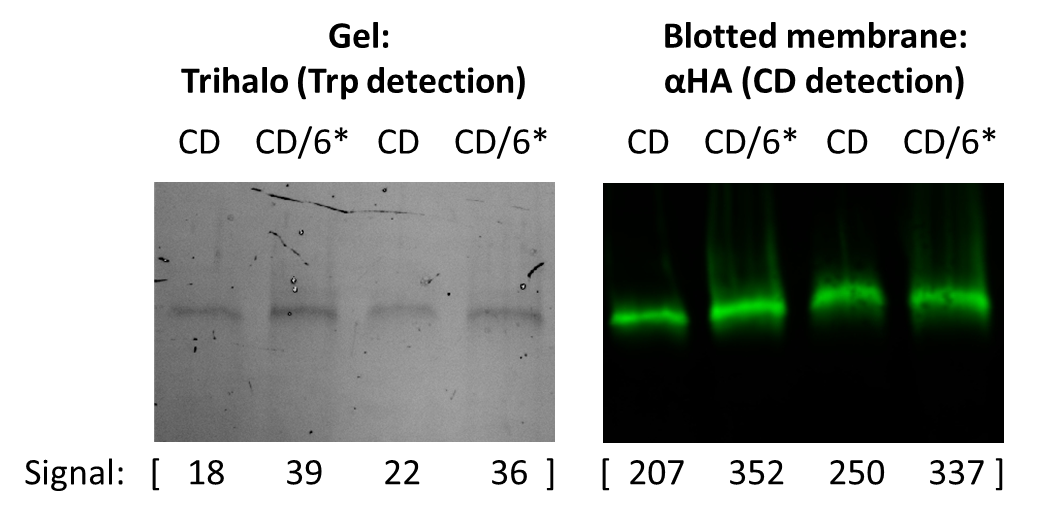
**

**Figure S9. Detection of tryptophan in purified CrCD protein from *C. reinhardtii* *CD* (positive control) and *CD/6** + *tCI* cell lines.** CrCD protein normally contains 7 tryptophans, all encoded by TGG; in *CD/6**, 6 of the 7 are mutated to TGA and are therefore reliant on tCI for translation.

Each sample was run in duplicate lanes on a Mini-PROTEAN Stain-Free Gel (Bio-Rad). The trihalo compounds in these gels react specifically with tryptophan residues in proteins to produce fluorescence in a UV-induced reaction. After UV activation, band intensities were quantified from the gel (left) to measure tryptophan using a Bio-Rad ChemiDoc XRS System. The gel was then blotted onto a membrane, which was probed with anti-HA antibodies (right) to measure the relative amount of purified CrCD protein loaded from each cell line (standard primary and secondary antibodies and detection, see Methods). After adjusting the shown trihalo signals to account for the heavier loading of the CD/6* protein, the trihalo signal ratio for CD:CD/6* is 1:1.2, i.e. there are approximately equal levels of tryptophan in both proteins. This suggests that tCI has kept its specificity for tryptophanyl-tRNA synthetase.

**SUPPLEMENTARY TABLES**

**Table S1.** Primers used to alter *trnW_UCA_* to make variants 1 to 4.

| **Variant** | **Forward primer 5**′**-3**′ | **Reverse primer 5**′**-3**′ |
| --- | --- | --- |
| 1 | tcctacaggg**t**gtgtttttcctaatgtac | ggaaaaacac**a**ccctgtaggaattga |
| 2 (first mutation) | ttttacgtcc**a**tagttcagtcggtag | gactgaacta**t**ggacgtaaaatttgt |
| 2 (second mutation) | caattcctac**c**gggcgtgtttttcct | aaacacgccc**g**gtaggaattgaaccc |
| 3 | agtcggtaga**g**cgcaggttt**tca**aaac | aaaacctgcg**c**tctaccgactgaact |
| 4 (first mutation) | attttacgtc**a**ttagttcagtcggta | actgaactaa**t**gacgtaaaatttgtt |
| 4 (second mutation) | aattcctaca**t**ggcgtgtttttccta | aaaacacgcc**a**tgtaggaattgaacc |

**Table S2.** Conditions for *C. reinhardtii* induction experiments.

|  |  | **Induction phase conditions (see figures for temperatures)** | | | | | |
| --- | --- | --- | --- | --- | --- | --- | --- |
| **Figure** | **Growth phase temperature (°C)** | **Culture style**  **(all TAP liquid medium)** | **Culture density at induction (OD_750_)** | **Culture density at sampling time (OD_750_)** | **Light (µE/m^2^/s)** | **Shaking (rpm)** | **Length of induction (h)** |
| 2 | 35 | 2.5 ml in 12 well plates | 0.2 | 2.2-4.3 | 25 | 0 | 72 |
| 4a | 30 | 2.5 ml in 12 well plates | 0.2 | 2.2-4.3 | 50 | 0 | 72 |
| 4b/S4 | 35 | 450 ml, glass flasks in Algem photobioreactor | 2.1-2.3 | 2.1-3.1 | 200 | 120 | 0-72 range |
| 5b | 30 | 20 ml, glass flasks | 2.0 | 2.6-4.0 | 50 | 0 | 0-96 range |
| 5c/d | 35 | 20 ml, glass flasks | 1.8 | 1.9-2.4 | 50 | 0 | 0-96 range |
| S3 | 35 | 450 ml, glass flasks in Algem photobioreactor | 2.5-2.7 | 1.2-2.7 | 200 | 120 | 0-120 range |

**SUPPLEMENTARY SEQUENCE INFORMATION**

**1) Sequence of the original synthetic *trnW_UCA_* gene with 100 bp flanks (Young and Purton 2016)**

Colours represent the tRNA sequence (green), anticodon (blue), and MluI sites used for cloning (red). The -10 and -35 consensus promoter elements are in **bold**. The 100 bp flanks are copied from those around the native *trnW* gene and were included to ensure that any necessary promoter and pre-tRNA elements are present.

tcgatgcACGCGTtaacccatgattaacaactatatcaataaaatcaatttgtagtgaaatactctga**ttgaca**ttaaaataataccatgataaaaat**tataat**aacaaattttacgtccttagttcagtcggtagaacgcaggttttcaaaacctgatgtcgtgggttcaattcctacagggcgtgtttttcctaatgtactttgttgtaaaagtggctggtttaacctttttaggtttcggattgaacaataatggcagttaagagtcactaaagctgctgtatagACGCGTtcgatgc

**2) Sequences of the variant *trnW_UCA_* genes tested in Figure 1**

All use the same 100 bp flanks as above (not shown). Introduced mutations are shown in black. Anticodon is in blue. Variant 1 was found to be temperature-sensitive and was renamed tCI, for use in the CITRIC system.

Original acgtccttagttcagtcggtagaacgcaggttttcaaaacctgatgtcgtgggttcaattcctacagggcgtg

Variant 1 acgtccttagttcagtcggtagaacgcaggttttcaaaacctgatgtcgtgggttcaattcctacagggtgtg

Variant 2 acgtccatagttcagtcggtagaacgcaggttttcaaaacctgatgtcgtgggttcaattcctaccgggcgtg

Variant 3 acgtccttagttcagtcggtagagcgcaggttttcaaaacctgatgtcgtgggttcaattcctacagggcgtg

Variant 4 acgtcattagttcagtcggtagaacgcaggttttcaaaacctgatgtcgtgggttcaattcctacatggcgtg

**3) Sequence of *CD* gene, encoding CrCD, showing position of TGG to TGA mutations introduced at tryptophan codons in the CD/2*, CD/4* and CD/6* versions (NB. these are cumulative).**

ATGTCTAACAACGCTTTACAAACAATTATTAACGCTCGTTTACCAGGTGAAGAAGGTTTATGACAAATTCACTTACAAGACGGTAAAATTTCAGCTATTGATGCTCAATCTGGTGTAATGCCAATTACTGAAAACTCTTTAGATGCTGAACAAGGTTTAGTTATTCCACCATTCGTTGAACCACACATTCACTTAGATACTACACAAACAGCTGGTCAACCAAACTGAAACCAATCAGGTACTTTATTTGAAGGTATTGAGCGTTGAGCTGAACGTAAAGCTTTATTAACACACGACGACGTTAAACAACGTGCTTGACAAACATTAAAATGACAAATTGCTAACGGTATTCAACACGTACGTACTCACGTAGACGTTTCTGATGCTACTTTAACAGCTTTAAAAGCTATGTTAGAAGTTAAACAAGAAGTAGCTCCATGAATTGACTTACAAATTGCTGCTTTCCCACAAGAAGGTATTTTATCATACCCAAACGGTGAAGCTTTATTAGAAGAAGCTTTACGTTTAGGTGCTGATGTTGTTGGTGCTATTCCACACTTCGAATTTACACGTGAATATGGTGTTGAATCTTTACACAAAACATTTGCTTTAGCTCAAAAATATGATCGTTTAATTGATGTTCACTGTGACGAAATTGATGACGAACAATCACGTTTCGTTGAAACAGTAGCTGCTTTAGCTCACCACGAAGGTATGGGTGCTCGTGTTACTGCTTCACACACTACAGCTATGCACTCTTACAACGGTGCTTACACTTCTCGTTTATTCCGTTTATTAAAAATGTCTGGTATTAACTTCGTTGCTAACCCATTAGTAAACATTCACTTACAAGGTCGTTTCGATACTTACCCAAAACGTCGTGGTATTACACGTGTTAAAGAAATGTTAGAATCAGGTATTAATGTTTGTTTTGGTCACGACGACGTTTGTGGTCCTTGGTACCCTTTAGGTACTGCTAACATGTTACAAGTTTTACACATGGGTTTACACGTATGTCAATTAATGGGTTACGGTCAAATTAACGACGGTTTAAACTTAATTACTCACCACTCTGCTCGTACTTTAAACTTACAAGACTACGGTATTGCTGCTGGTAACTCAGCTAACTTAATTATTTTACCAGCTGAAAACGGTTTCGATGCTTTACGTCGTCAAGTTCCAGTACGTTACTCAGTTCGTGGTGGTAAAGTTATTGCTTCAACTCAACCAGCTCAAACAACTGTTTATTTAGAACAACCAGAAGCTATTGACTACAAACGTTACCCATACGATGTTCCAGATTACGCTTAATAA

**4) Conceptually translated sequence of CrCD, with highlighting as above.**

HA tag is shown in red. Residues in turquoise show where the synthetic CrCD enzyme differs from natural *E. coli* CodA for improved substrate binding; see Young & Purton (2014)*.*

MSNNALQTIINARLPGEEGLWQIHLQDGKISAIDAQSGVMPITENSLDAEQGLVIPPFVEPHIHLDTTQTAGQPNWNQSGTLFEGIERWAERKALLTHDDVKQRAWQTLKWQIANGIQHVRTHVDVSDATLTALKAMLEVKQEVAPWIDLQIAAFPQEGILSYPNGEALLEEALRLGADVVGAIPHFEFTREYGVESLHKTFALAQKYDRLIDVHCDEIDDEQSRFVETVAALAHHEGMGARVTASHTTAMHSYNGAYTSRLFRLLKMSGINFVANPLVNIHLQGRFDTYPKRRGITRVKEMLESGINVCFGHDDVCGPWYPLGTANMLQVLHMGLHVCQLMGYGQINDGLNLITHHSARTLNLQDYGIAAGNSANLIILPAENGFDALRRQVPVRYSVRGGKVIASTQPAQTTVYLEQPEAIDYKRYPYDVPDYA

**5) Sequence of *TPS4* gene, showing position of TGG to TGA mutations introduced at tryptophan codons in the TPS4/2* and TPS4/4* versions (NB. these are cumulative).**

This is a version of the *Abies balsamea TPS4* gene that has been codon optimised for the *C. reinhardtii* chloroplast and has a C-terminal HA tag added. Gene (without TGA mutations) provided by Julie Zedler, University of Kent.

ATGAACCGTGAATTCCCTCCATCTTTCTGAAACAACGATATTATTAACTCAATTACTGCTTCACACAAAGTACAAACAGGTGACCGTAAACGTATTCAAACATTAATTTCAGAAATTAAAAACGTATTCAACTCAATGGGTGATGGTGAAACTTCTCCATCAGCTTACGATACTGCTTGAGTTGCTCGTATTCCAGCTGTAGATGGTTCAGAACAACCACAATTCCCACAAACATTAGAATGGATTTTACAAAACCAATTAAAAGATGGTTCTTGGGGTGAAGAATTCTACTTCTTAGCTTACGATCGTTTATTAGCTACTTTAGCTTGTATTATTACTTTAACAATTTGACGTACAGGTAACGTTCAATTACACAAAGGTATTGAATTCTTCCGTAAACAAGTTGTTCGTATGGATGATGAAGCTGATAACCACCGTCCATCAGGTTTCGAAATTGTATTCCCTGCTATGTTAAACGAAGCTAAATCATTAGGTTTAGATTTACCATATGAATTACCATTCATTGAACAAATGGTTAAAAAACGTGAAGCTAAATTAAAAATGATTACTACAAACGTTTTATACACTATTCAAACAACTTTATTATACAGTTTAGAAGGTTTACACGAAATTGTAGACTTCGATAAAATTATTAAATTACAATCTAAAGATGGTTCTTTCTTAGGTTCTCCAGCTTCAACTGCTGCTGTATTCATGCAAACAGGTAACACTAAATGTTTAGAATTCTTAGAATTCGTTTTACGTAAATTCCGTAACCACGTACCATCTGACTACCCATTAGATTTATTCGAACGTTTATGAGTTGTAGATACTGTTGAACGTTTAGGTATTGACCGTCACTTCAAAAAAGAAATTAAAGATGCTTTAGATTACGTATACAGCTGTTGGGATGAACGTGGTATTGGTTGGGCTAAAGATTCTCCAATTGCTGATATTGATGATACTGCTATGGGTTTACGTATTTTACGTTTACACGGTTACAACGTTTCTCCAGATGTTTTAAAAACATTCAAAGATGAAAACGGTGAATTCTTCTGTTTCATGGGTCAAACACAACGTGGTGTTACTGACATGTTAAACGTATACCGTTGTTCACAAGTAGCTTTCCCTGGTGAAACTATTATGGAAGAAGCTAAATTATGTACAGAACGTTACTTACGTAACGCTTTAGAAAACGCTGATGCTTTCGATAAATGGGCTATTAAAAAAAACATTCGTGGTGAAGTTGAATACGCTTTAAAATACCCATGGCACCGTTCAATGCCACGTTTAGAAGTTCGTTCATACATTGGTAACTACGGTCCAAACGATGTTTGGTTAGGTAAATCATTATACATGATGCCATACATTTCTAACGAAAAATATTTAGAATTAGCTAAATTAGATTTCAACTCAGTACAATCATTACACCAAGAAGAAATTCGTGAATTAGTTCGTTGGTGTAAATCATCTGGTTTTACTGAATTAAAATTTACACGTGACCGTGTAGTTGAAACTTACTTTGCTGTAGCTTCTTCTATGTTCGAACCAGAATTCTCAACTTGTCGTGCTGTTTACACTAAAATTTCAGTATTATTAGTTATTTTAGATGACTTATACGATGGTTACGGTTCTCCAGATGAAATTAAATTATTCTCAGAAGCTGTTAAACGTTGGGATTTATCTTTATTAGAACAAATGCCAGATCACATGAAAATTTGTTTCTTAGGTTTATACAACACAGTAAACGAAGTAGCTGAAGAAGGTCGTAAAACACAAGGTCACGACGTATTAGGTTACATTCGTAACTTATGGGAAATTCAATTAGCTGCTTTCACTCGTGAAGCTGAATGGTCACAAGGTAAATATGTTCCATCTTTCGATGAATACATTGAAAACGCTCAAGTATCAATTGGTGTAGCTACTATTTTATTAATTACTATTTTATTTACTGAAGAAGATGATATTTTATCTCACATTGACTACGGTTCAAAATTCTTACGTTTAGCTTCATTAACAGCTCGTTTAGCTAACGATATTAAAACATACCAAGAAGAACGTGCTCACGGTGAAGTTGTTTCTGCTATTCAATGTTACATGAAAGACCGTCCAGAAATTACTGAAGAAGAAGCTTTAAAATATGTTTACGGTCGTATGGTTAACGATTTAGCTGAATTAAACTCAGAATACTTAAAATCAAACGAAATGCCACAAAACTGTAAACGTTTAGTATTTGATACTGCTCGTGTAGCTCAATTATTTACTATGGAAGGTGATGGTTTAACATACAGTGATACTATGGAAATTAAAGAACACATTAAAAAATGTTTATTTGAACCAGCTACTTACCCATACGATGTTCCAGATTACGCTTAATAA

**6) Conceptually translated sequence of TPS4, with highlighting as above.**

HA tag is shown in red.

MNREFPPSFWNNDIINSITASHKVQTGDRKRIQTLISEIKNVFNSMGDGETSPSAYDTAWVARIPAVDGSEQPQFPQTLEWILQNQLKDGSWGEEFYFLAYDRLLATLACIITLTIWRTGNVQLHKGIEFFRKQVVRMDDEADNHRPSGFEIVFPAMLNEAKSLGLDLPYELPFIEQMVKKREAKLKMITTNVLYTIQTTLLYSLEGLHEIVDFDKIIKLQSKDGSFLGSPASTAAVFMQTGNTKCLEFLEFVLRKFRNHVPSDYPLDLFERLWVVDTVERLGIDRHFKKEIKDALDYVYSCWDERGIGWAKDSPIADIDDTAMGLRILRLHGYNVSPDVLKTFKDENGEFFCFMGQTQRGVTDMLNVYRCSQVAFPGETIMEEAKLCTERYLRNALENADAFDKWAIKKNIRGEVEYALKYPWHRSMPRLEVRSYIGNYGPNDVWLGKSLYMMPYISNEKYLELAKLDFNSVQSLHQEEIRELVRWCKSSGFTELKFTRDRVVETYFAVASSMFEPEFSTCRAVYTKISVLLVILDDLYDGYGSPDEIKLFSEAVKRWDLSLLEQMPDHMKICFLGLYNTVNEVAEEGRKTQGHDVLGYIRNLWEIQLAAFTREAEWSQGKYVPSFDEYIENAQVSIGVATILLITILFTEEDDILSHIDYGSKFLRLASLTARLANDIKTYQEERAHGEVVSAIQCYMKDRPEITEEEALKYVYGRMVNDLAELNSEYLKSNEMPQNCKRLVFDTARVAQLFTMEGDGLTYSDTMEIKEHIKKCLFEPATYPYDVPDYA

**7) Upstream element used for expressing TPS4.**

16S rRNA promoter region is underlined, with -35 and -10 promoter elements in yellow and 16S transcriptional start in pink (i.e. the 16S region used extends beyond the promoter). *PsaA* exon 1 5'UTR is in **bold**. This was immediately followed by the *TPS4* start codon.

GGCAGGCAACAAATTTATTTATTGTCCCGTAAGGGGAAGGGGAAAACAATTATTATTTTACTGCGGAGCAGCTTGTTATTGAAATTTTATTAAAAAAAAAATAAAAATTTGACAAAAAAAAATAAAAAAGTTAAATTAAAAACACTGGGAATGTTCTACATCATAAAAATCAAAAGGGTTTAAAATCCCGACAAAATTTAAACTTTAAAGAGT**atgatgtaaaaaaaactatttgtctaatttaataaccatgcattttttatgaacacataataattaaaagcgttgctaatggtgtaaataatgtatttattaaattaaataattgttattataaggagaaatcc**

**8) Sequence of plasmid pWUCA3.**

The *trnW_UCA_* region is colour coded as in Sequences 1 and 2 above. *PsaA* exon 3 (forward orientation) is highlighted in turquoise. The *aadA* spectinomycin resistance gene (reverse orientation) is highlighted in pink and its promoter/UTRs in green. Ampicillin resistance gene for selection in *E. coli* is in orange text. Plasmid pWUCA3 is identical to pWUCA1 (Young & Purton 2016) except the single base change that converts the original tRNA^Trp^-UCA (constitutive) to tCI (cold-inducible).

CCCATCAAGCTTATCGATACCGTCGACCTCGAGGGGGGGCCCGGTACCCAATTCGCCCTATAGTGAGTCGTATTACAATTCACTGGCCGTCGTTTTACAACGTCGTGACTGGGAAAACCCTGGCGTTACCCAACTTAATCGCCTTGCAGCACATCCCCCTTTCGCCAGCTGGCGTAATAGCGAAGAGGCCCGCACCGATCGCCCTTCCCAACAGTTGCGCAGCCTGAATGGCGAATGGAAATTGTAAGCGTTAATATTTTGTTAAAATTCGCGTTAAATTTTTGTTAAATCAGCTCATTTTTTAACCAATAGGCCGAAATCGGCAAAATCCCTTATAAATCAAAAGAATAGACCGAGATAGGGTTGAGTGTTGTTCCAGTTTGGAACAAGAGTCCACTATTAAAGAACGTGGACTCCAACGTCAAAGGGCGAAAAACCGTCTATCAGGGCGATGGCCCACTACGTGAACCATCACCCTAATCAAGTTTTTTGGGGTCGAGGTGCCGTAAAGCACTAAATCGGAACCCTAAAGGGAGCCCCCGATTTAGAGCTTGACGGGGAAAGCCGGCGAACGTGGCGAGAAAGGAAGGGAAGAAAGCGAAAGGAGCGGGCGCTAGGGCGCTGGCAAGTGTAGCGGTCACGCTGCGCGTAACCACCACACCCGCCGCGCTTAATGCGCCGCTACAGGGCGCGTCAGGTGGCACTTTTCGGGGAAATGTGCGCGGAACCCCTATTTGTTTATTTTTCTAAATACATTCAAATATGTATCCGCTCATGAGACAATAACCCTGATAAATGCTTCAATAATATTGAAAAAGGAAGAGTATGAGTATTCAACATTTCCGTGTCGCCCTTATTCCCTTTTTTGCGGCATTTTGCCTTCCTGTTTTTGCTCACCCAGAAACGCTGGTGAAAGTAAAAGATGCTGAAGATCAGTTGGGTGCACGAGTGGGTTACATCGAACTGGATCTCAACAGCGGTAAGATCCTTGAGAGTTTTCGCCCCGAAGAACGTTTTCCAATGATGAGCACTTTTAAAGTTCTGCTATGTGGCGCGGTATTATCCCGTATTGACGCCGGGCAAGAGCAACTCGGTCGCCGCATACACTATTCTCAGAATGACTTGGTTGAGTACTCACCAGTCACAGAAAAGCATCTTACGGATGGCATGACAGTAAGAGAATTATGCAGTGCTGCCATAACCATGAGTGATAACACTGCGGCCAACTTACTTCTGACAACGATCGGAGGACCGAAGGAGCTAACCGCTTTTTTGCACAACATGGGGGATCATGTAACTCGCCTTGATCGTTGGGAACCGGAGCTGAATGAAGCCATACCAAACGACGAGCGTGACACCACGATGCCTGTAGCAATGGCAACAACGTTGCGCAAACTATTAACTGGCGAACTACTTACTCTAGCTTCCCGGCAACAATTAATAGACTGGATGGAGGCGGATAAAGTTGCAGGACCACTTCTGCGCTCGGCCCTTCCGGCTGGCTGGTTTATTGCTGATAAATCTGGAGCCGGTGAGCGTGGGTCTCGCGGTATCATTGCAGCACTGGGGCCAGATGGTAAGCCCTCCCGTATCGTAGTTATCTACACGACGGGGAGTCAGGCAACTATGGATGAACGAAATAGACAGATCGCTGAGATAGGTGCCTCACTGATTAAGCATTGGTAACTGTCAGACCAAGTTTACTCATATATACTTTAGATTGATTTAAAACTTCATTTTTAATTTAAAAGGATCTAGGTGAAGATCCTTTTTGATAATCTCATGACCAAAATCCCTTAACGTGAGTTTTCGTTCCACTGAGCGTCAGACCCCGTAGAAAAGATCAAAGGATCTTCTTGAGATCCTTTTTTTCTGCGCGTAATCTGCTGCTTGCAAACAAAAAAACCACCGCTACCAGCGGTGGTTTGTTTGCCGGATCAAGAGCTACCAACTCTTTTTCCGAAGGTAACTGGCTTCAGCAGAGCGCAGATACCAAATACTGTCCTTCTAGTGTAGCCGTAGTTAGGCCACCACTTCAAGAACTCTGTAGCACCGCCTACATACCTCGCTCTGCTAATCCTGTTACCAGTGGCTGCTGCCAGTGGCGATAAGTCGTGTCTTACCGGGTTGGACTCAAGACGATAGTTACCGGATAAGGCGCAGCGGTCGGGCTGAACGGGGGGTTCGTGCACACAGCCCAGCTTGGAGCGAACGACCTACACCGAACTGAGATACCTACAGCGTGAGCTATGAGAAAGCGCCACGCTTCCCGAAGGGAGAAAGGCGGACAGGTATCCGGTAAGCGGCAGGGTCGGAACAGGAGAGCGCACGAGGGAGCTTCCAGGGGGAAACGCCTGGTATCTTTATAGTCCTGTCGGGTTTCGCCACCTCTGACTTGAGCGTCGATTTTTGTGATGCTCGTCAGGGGGGCGGAGCCTATGGAAAAACGCCAGCAACGCGGCCTTTTTACGGTTCCTGGCCTTTTGCTGGCCTTTTGCTCACATGTTCTTTCCTGCGTTATCCCCTGATTCTGTGGATAACCGTATTACCGCCTTTGAGTGAGCTGATACCGCTCGCCGCAGCCGAACGACCGAGCGCAGCGAGTCAGTGAGCGAGGAAGCGGAAGAGCGCCCAATACGCAAACCGCCTCTCCCCGCGCGTTGGCCGATTCATTAATGCAGCTGGCACGACAGGTTTCCCGACTGGAAAGCGGGCAGTGAGCGCAACGCAATTAATGTGAGTTAGCTCACTCATTAGGCACCCCAGGCTTTACACTTTATGCTTCCGGCTCGTATGTTGTGTGGAATTGTGAGCGGATAACAATTTCACACAGGAAACAGCTATGACCATGATTACGCCAAGCTCGAAATTAACCCTCACTAAAGGGAACAAAAGCTGGAGCTCCACCGCGGTGGCGGCCGCTctaggtatatacattcaccctttaaggctacccggcagttagttacggcttacgttccataaaatattggcatattttataaattattttatagatcatatattttgtaaatatataatattacgcataacacatttatttaaaaacatataatattacgcataacacatttatttaaaaacagcaaaaacttgcgtcaaatccctataggatattttttatttatggcacgagcaggtttacacgctccgtcaggacgccggcacgtagttggaaagtatgtcccccttgcccggaaggggaaaggaggagacaaatttatttattgtatataaatagcatgactttccaagcgagttaacataaacaaactgcgcgagttaacataaacaaacttcctctctggggaggcatggcagcaaatggcaccctaaatacatgcgcttttaacacagataattataaacaagcatagcgttaaatctgctaccttggattaaatcattagaaagaatttgagccgtgtgcagtgaaaattgcatgcacggctcttaaggtttaaataaatttttaaagaagaaaatttaactcctaactatgtacttccatggtgcacgtttttcaaactatgaagcttggttaagtgaccctactcacattaaaccaagtgctcaagtagtatggcctattgtaggtcaagaaattttaaacggtgatgtaggtggtggtttccaaggtattcaaattacttctggtttcttccaattatggcgtgctagtggtattactagtgaattacaactttatactacagcaattggtggtttagtaatggctgctgcaatgttctttgctggttggttccactaccacaaagctgctccaaaactagaatggttccaaaacgttgaatcaatgttaaaccaccacttaggtggtcttcttggtttaggtagtttagcttgggctggtcaccaaattcacgtttctttaccagtaaacaaattattagatgctggtgtagatccaaaagaaattccacttcctcatgatttattattaaatcgtgctattatggctgacttatacccaagttttgctaaaggtattgctcctttctttactttaaactggagtgaatacagtgatttcttaacatttaaaggtggtttaaaccctgttactggtggtctttggttaagtgatactgctcaccaccacgtagctattgctgtattattcttagtagctggtcacatgtatcgtactaactggggtattggtcacagtatgaaagaaattttagaagctcaccgtggtccatttacaggtgaaggtcacgttggtttatatgaaattttaacaacttcttggcatgcacaattagctattaacttagctttatttggttcgttatcaattattgtagctcaccacatgtacgcaatgcctccatacccttatttagctactgattacggtacacaattatcattatttacacaccacacatggattggtggtttctgtattgttggtgctggtgctcacgcagctattttcatggttcgtgactacgatcctactaataactacaacaacttattagaccgtgtaattcgtcaccgtgatgctattatttctcacttaaactgggtttgtattttcttaggtttccacagctttggtttatacatccacaacgatacaatgagtgctttaggtcgtcctcaagacatgttctcagatactgctatccaacttcaaccagtatttgctcaatggattcaaaatacacacttcttagctccacaattaacagcaccaaatgctttagctgctacaagtttaacttggggtggtgagctaggtgctcatggcggtaaagtagctatgatgcctatttctttaggtacttctgactttatggttcaccacattcacgctttcacaattcacgtaactgtgttaattcttctgaaaggtgttttatttgctcgtagctctcgtcttatcccagataaagctaacttaggtttccgtttcccttgtgacggtcctggtcgtggcggtacttgtcaggtttctgcttgggaccacgtattcttaggtcttttctggatgtacaacagcttatcaattgtaattttccacttcagctggaagatgcaatctgatgtttggggtacggttacagcttctggtgtttctcacattactggtggtaactttgcacaaagcgctaacacaatcaacggttggttacgtgacttcttatgggcacaatcatcacaagtaatccaatcatacggttcagctctatctgcttatggtttaattttcttaggtgctcacttcgtatgggcattctcgttaatgttcttattctctggtcgtggttactggcaagaacttatcgaatcaattgtatgggctcacaacaaacttaaagttgcacctgcaattcaaccacgtgctttaagtattactcaaggtcgtgctgttggtgtagctcactaccttttaggtggtattgctactacatggtcgttcttcttagcacgtatcatttctgtaggttaacatttaatactttttaatacatatatgcctaagtttatctttaaagataaacttagccatatgtgttaagttatctaacaaggttacctttttatttctctttagatatataaacattaaaaactaccgtgatcgttacactttagataactggaagggggaaaaatcatgtattcgctggaaggcgcacctcctactgcctactgcgcagcattaaaatgctgtagatattggtatcttacaaaggacagtagtacacaattaa**ACGCGT**taacccatgattaacaactatatcaataaaatcaatttgtagtgaaatactctga**ttgaca**ttaaaataataccatgataaaaat**tataat**aacaaattttacgtccttagttcagtcggtagaacgcaggttt**tca**aaacctgatgtcgtgggttcaattcctacaggg**t**gtgtttttcctaatgtactttgttgtaaaagtggctggtttaacctttttaggtttcggattgaacaataatggcagttaagagtcactaaagctgctgtatag**ACGCGT**tggaatatttatattttcattagagAATTgggtaccgagctccaccgcggtggcggccgctctagctagaactagtggatcgcactctaccgattgagttacatccgctttagtatgttactatttcttttattataacttataaaatataatacataaagataaattctataataaaaagctaagattttatttttctggcacatcgtaatttataaagacaggcaaatttaaacaaaagataactttagaacttaattttaaaaatgtaaaatgatgtttaggtatttaacctaaacaccataaaaataaaaacgatgtttatgctattcacataaacatcatgaaaaataaaaattaaagtttgtcaatagtatcaaattcgaatttaatttctttccaaacttcacatgcagcagcaagttctggagaccatttacaagctgaacgaattacgtcgccaccttcacgagcaaggtcacgaccttcgttacgagcttgagtacaagcttgcatgcctgcagTTATTTGCCAACTACCTTAGTGATCTCGCCTTTCACGTAGTGGACAAATTCTTCCAACTGATCTGCGCGCGAGGCCAAGCGATCTTCTTCTTGTCCAAGATAAGCCTGTCTAGCTTCAAGTATGACGGGCTGATACTGGGCCGGCAGGCGCTCCATTGCCCAGTCGGCAGCGACATCCTTCGGCGCGATTTTGCCGGTTACTGCGCTGTACCAAATGCGGGACAACGTAAGCACTACATTTCGCTCATCGCCAGCCCAGTCGGGCGGCGAGTTCCATAGCGTTAAGGTTTCATTTAGCGCCTCAAATAGATCCTGTTCAGGAACCGGATCAAAGAGTTCCTCCGCCGCTGGACCTACCAAGGCAACGCTATGTTCTCTTGCTTTTGTCAGCAAGATAGCCAGATCAATGTCGATCGTGGCTGGCTCGAAGATACCTGCAAGAATGTCATTGCGCTGCCATTCTCCAAATTGCAGTTCGCGCTTAGCTGGATAACGCCACGGAATGATGTCGTCGTGCACAACAATGGTGACTTCTACAGCGCGGAGAATCTCGCTCTCTCCAGGGGAAGCCGAAGTTTCCAAAAGGTCGTTGATCAAAGCTCGCCGCGTTGTTTCATCAAGCCTTACGGTCACCGTAACCAGCAAATCAATATCACTGTGTGGCTTCAGGCCGCCATCCACTGCGGAGCCGTACAAATGTACGGCCAGCAACGTCGGTTCGAGATGGCGCTCGATGACGCCAACTACCTCTGATAGTTGAGTTGATACTTCGGCGATAACCGCTTCACGAGCCATggacattttcacttctggagtgtattgttcaattaaatctttaataagattactaagttcttctggagtacgcattgccataaaaaagaaaaaataaataaaagattaaaaaagtttatttttaaaatctttctcgagaattttaaataagtttaaaattcaacaaaaatagtgagtggtaagatcacttgttaacaaaagtaatggttcacccttgtcatatttaaatactaaaattcatttgcccgaagaggacaaatttatttattgcattaaaatccctaagtttacttgcccgtaaggggaagggggggacgtccacaggcgtcgtaagcaactaaagtttatgacgccgattgctttgttaggaaaatataaatatcccataagaaaaggtcctttaaaggttttatggactaaataaaaaagatagcataagcattaaaatcatgcaaattaaaaaaaaggtaaatgtatttataaaaaggtaaatgtatttatatagtatttatattatagcataataataaatatatttataaattgattgttcttagagctaaaagagaagaacaatgggtttataggtattttgagatccagttataaaaatgacttttgacgtttatggtatataaacactgcctctaataaagtcatcgataagcttgatcccatatagccaatggcttaaggagtgtcataggaataactagtcatgcacattttcctaaaaatctaaaatgttattagaagctatacaaaaattaaaaattatgttattatatatttattaaaatttagacttatcacggggatatggcggaatggtagacgctacggacttaaaatccgttcttgtgcgaacaaggtgagggttcaagtccctctttccccatttataaataaaaatcaagtcaatattttaacattgtaaactaaaaaataagtgtaaaataactagggtaaatagttaagaattggtagtttttgaactaaacagtgacataaaactggttaaaagacatccctgtaagagaaatgcatatggtgaattacacaataaatttaaaaataaagctctgacaagcctcttcccctttgcgatatacatgctctgttagatataattctgcctaagttcctaacaaagggtttactttttctcggggaatggttttgctactttgttttaaatctttttcaaagaatgtcaatctcctataaagggaggaacattactcatggtctcacaattttaataaactagtttcttataatctattttataaaaataaatctaaatatatttcttatgaattttgttaatttagaacaaattgaaaattctttacgtaatgctactttttgcatgctttttttaacaacatttttatattggttttatactgcattttacagtacaaatcctcaacaaattataaatccattgtcattaacaaacattaaaactaattttacttatcctgacgggtttcaagcaaacaatattaatgtttctacaacttatttaccgattaaccctgtattaaataccgaaagagaagagcaacccgaagctaacggaacgaatgggctgttgggtgtttcttcattagtagtaaatttaaaatcaatagcaatcccacgcattatgatgggtgtttctaatttattattagtattattattacttgttcgttgggaaaaatcaggtcatttcccattaagtaatttatatgaatctttaatgtttttagcgtggtgttgtacatttttatatttattatattgtacaagttttactttattggttgaaaaaatgttaggttcattaattgcaccttgtagtttattaatgaatgcatttgctacttttagtttaccaaaagaaatgcaacaagcatcaccattagtaccagctttacaatcaaattggttaatgatgcatgttactgtaatgattattagttatgccacattaattattggatcgttattgtcgattttatttttgattttatttaaacacaaaaaaggtacacccaaaaagtatgataactttattaacaatttagatgcattaagttatcgcattattggattaggttttccttttttaactattgggattttatctggggctgtgtgggctaatgaagcatggggatcatattggagttgggatcc

**9) Sequence of plasmid pWUCA4**

The *trnW_UCA_* region is colour coded as in Sequences 1 and 2 above. The *psaA* exon 1 promoter and 5’ UTR are highlighted in pink and the SapI and SphI sites for inserting a gene of interest are yellow. The *psbH* gene (reverse orientation) is highlighted in turquoise. Ampicillin resistance gene for selection in *E. coli* is in orange text. Plasmid pWUCA4 is identical to pWUCA2 (Young & Purton 2016) except the single base change that converts the original tRNA^Trp^-UCA (constitutive) to tCI (cold-inducible).

AGCTTGGCACTGGCCGTCGTTTTACAACGTCGTGACTGGGAAAACCCTGGCGTTACCCAACTTAATCGCCTTGCAGCACATCCCCCTTTCGCCAGCTGGCGTAATAGCGAAGAGGCCCGCACCGATCGCCCTTCCCAACAGTTGCGCAGCCTGAATGGCGAATGGCGCCTGATGCGGTATTTTCTCCTTACGCATCTGTGCGGTATTTCACACCGCATATGGTGCACTCTCAGTACAATCTGCTCTGATGCCGCATAGTTAAGCCAGCCCCGACACCCGCCAACACCCGCTGACGCGCCCTGACGGGCTTGTCTGCTCCCGGCATCCGCTTACAGACAAGCTGTGACCGTCTCCGGGAGCTGCATGTGTCAGAGGTTTTCACCGTCATCACCGAAACGCGCGAGACGAAAGGGCCTCGTGATACGCCTATTTTTATAGGTTAATGTCATGATAATAATGGTTTCTTAGACGTCAGGTGGCACTTTTCGGGGAAATGTGCGCGGAACCCCTATTTGTTTATTTTTCTAAATACATTCAAATATGTATCCGCTCATGAGACAATAACCCTGATAAATGCTTCAATAATATTGAAAAAGGAAGAGTATGAGTATTCAACATTTCCGTGTCGCCCTTATTCCCTTTTTTGCGGCATTTTGCCTTCCTGTTTTTGCTCACCCAGAAACGCTGGTGAAAGTAAAAGATGCTGAAGATCAGTTGGGTGCACGAGTGGGTTACATCGAACTGGATCTCAACAGCGGTAAGATCCTTGAGAGTTTTCGCCCCGAAGAACGTTTTCCAATGATGAGCACTTTTAAAGTTCTGCTATGTGGCGCGGTATTATCCCGTATTGACGCCGGGCAAGAGCAACTCGGTCGCCGCATACACTATTCTCAGAATGACTTGGTTGAGTACTCACCAGTCACAGAAAAGCATCTTACGGATGGCATGACAGTAAGAGAATTATGCAGTGCTGCCATAACCATGAGTGATAACACTGCGGCCAACTTACTTCTGACAACGATCGGAGGACCGAAGGAGCTAACCGCTTTTTTGCACAACATGGGGGATCATGTAACTCGCCTTGATCGTTGGGAACCGGAGCTGAATGAAGCCATACCAAACGACGAGCGTGACACCACGATGCCTGTAGCAATGGCAACAACGTTGCGCAAACTATTAACTGGCGAACTACTTACTCTAGCTTCCCGGCAACAATTAATAGACTGGATGGAGGCGGATAAAGTTGCAGGACCACTTCTGCGCTCGGCCCTTCCGGCTGGCTGGTTTATTGCTGATAAATCTGGAGCCGGTGAGCGTGGGTCTCGCGGTATCATTGCAGCACTGGGGCCAGATGGTAAGCCCTCCCGTATCGTAGTTATCTACACGACGGGGAGTCAGGCAACTATGGATGAACGAAATAGACAGATCGCTGAGATAGGTGCCTCACTGATTAAGCATTGGTAACTGTCAGACCAAGTTTACTCATATATACTTTAGATTGATTTAAAACTTCATTTTTAATTTAAAAGGATCTAGGTGAAGATCCTTTTTGATAATCTCATGACCAAAATCCCTTAACGTGAGTTTTCGTTCCACTGAGCGTCAGACCCCGTAGAAAAGATCAAAGGATCTTCTTGAGATCCTTTTTTTCTGCGCGTAATCTGCTGCTTGCAAACAAAAAAACCACCGCTACCAGCGGTGGTTTGTTTGCCGGATCAAGAGCTACCAACTCTTTTTCCGAAGGTAACTGGCTTCAGCAGAGCGCAGATACCAAATACTGTCCTTCTAGTGTAGCCGTAGTTAGGCCACCACTTCAAGAACTCTGTAGCACCGCCTACATACCTCGCTCTGCTAATCCTGTTACCAGTGGCTGCTGCCAGTGGCGATAAGTCGTGTCTTACCGGGTTGGACTCAAGACGATAGTTACCGGATAAGGCGCAGCGGTCGGGCTGAACGGGGGGTTCGTGCACACAGCCCAGCTTGGAGCGAACGACCTACACCGAACTGAGATACCTACAGCGTGAGCTATGAGAAAGCGCCACGCTTCCCGAAGGGAGAAAGGCGGACAGGTATCCGGTAAGCGGCAGGGTCGGAACAGGAGAGCGCACGAGGGAGCTTCCAGGGGGAAACGCCTGGTATCTTTATAGTCCTGTCGGGTTTCGCCACCTCTGACTTGAGCGTCGATTTTTGTGATGCTCGTCAGGGGGGCGGAGCCTATGGAAAAACGCCAGCAACGCGGCCTTTTTACGGTTCCTGGCCTTTTGCTGGCCTTTTGCTCACATGTTCTTTCCTGCGTTATCCCCTGATTCTGTGGATAACCGTATTACCGCCTTTGAGTGAGCTGATACCGCTCGCCGCAGCCGAACGACCGAGCGCAGCGAGTCAGTGAGCGAGGAAGCAATTCgaatccgcgttttctccgtgaaagggaggtgtcctaggcctctagacgatgggggctttttgttatattttactaaatatatattataattaaaaaaaattgaattgtcaatttttaatgtacacttagttgaaagtgcccctgtccccttggccatatttaacagaagttatttataacgcagctgttttttggagtctataaatttataacatcagttactatggatttccctttagttttatggcctaggacgtccccttccccttcgatgctggaggcatccttttacgggacaataaataaatttgttgcctcgcctatcggctaacaagttccttcggagtatataaatataggatgttaatactgctataaactttagttgcccaatatttatattaggacgccagtggcagtggtaccgccactgcctgcttcgcagtatataaatataggcagttggcaggcaactgccactgacgtcctattttaatactcccaagtttacttgcctaggcagttggcaggcaacaaatttatttattgtccactaaaatttatttgcccgaaggggacgtccactaaaatttatttacccgaaggggacgtcctaatataaatatggggatgtcaatgctccgttaggaagtaactaacgtttttcaaataaattttatcccggagggaagtaggcagtagcccgccactgtcatcctttaagtggatctctcgtcaggcaatttgcttacacctttaaattaaaaattaaatttaaagaaaagtgagctattaACGCGTtaacccatgattaacaactatatcaataaaatcaatttgtagtgaaatactctga**ttgaca**ttaaaataataccatgataaaaat**tataat**aacaaattttacgtccttagttcagtcggtagaacgcaggttt**tca**aaacctgatgtcgtgggttcaattcctacaggg**t**gtgtttttcctaatgtactttgttgtaaaagtggctggtttaacctttttaggtttcggattgaacaataatggcagttaagagtcactaaagctgctgtatagACGCGTaagctttcttaattcaacatttttaagtaaatactgtttaatgttatacttttacgaatacacatatggtaaaaaataaaacaatatctttaaaataagtaaaaataatttgtaaaccaataaaaaatatatttatggtataatataacatatgatgtaaaaaaaactatttgtctaatttaataaccatgcattttttatgaacacataataattaaaagcgttgctaatggtgtaaataatgtatttattaaattaaataattgttattataaggagaaatccATGggaagagctactccatggatcctctagagtcgacctgcaggcatgcaagcttgtactcaagctcgtaacgaaggtcgtgaccttgctcgtgaaggtggcgacgtaattcgttcagcttgtaaatggtctccagaacttgctgctgcatgtgaagtttggaaagaaattaaattcgaatttgatactattgacaaacttTAAtttttatttttcatgatgtttatgtgaatagcataaacatcgtttttatttttatggtgtttaggttaaatacctaaacatcattttacatttttaaaattaagttctaaagttatcttttgtttaaatttgcctgtctttataaattacgatgtgccagaaaaataaaatcttagctttttattatagaatttatctttatgtattatattttataagttataataaaagaaatagtaacatactaaagcggatgtaGCGCGTttatcttaacggaaggccagtggcagtggcggtgccactgccgaatataaatatggttgagttgcttagtttaccttagcgaaaagaagacttagcagctagccttaacaaacagttttatattttatgtttgtgttaaataaaaTTAAGAAACTTTAGCTAAAGTTTCCCAACTCATAGAAACGTCATCTAAAATTAAAGAACTGTTGTAAATTTCTAAAATGATTAATAAGAATGCTGCAAATAAAAGGATAAATACAGCCATTAAAACAGTTGTACCCCAGCCTGGTAATACTTTACCTGCTTCTGAGTTAAGTGGACGTAATAAAGTACCTAATGGTGTAACTAAACCAGGTTCTTGGAAGTCTGAATTTACTTTTGATGGTTTAGCTTTAGAAGTTCCTGTTGCCATaattgattaaatgaattaagcgttattagcgctattttatttactttctgtaaaaaataaggaaaatattcttcagtgcattccctctcaggattataaatactctgaggataacgttctctcgtcaaggggttgcttcttgtgagtatagaaacctactagcacaagaaataaattgcataaaaatgtatttacctaggaccgcagtaggcagtcccttttccccttcagaactgcctgctttaaaagaatgaaaaaactgccttgtctggtaagtaaaactctttaattactcactaaagacgatcttagaagttctttgttcattttttatttaatataatatttgttatataaaaattaaataatttttaattaatgtttaactttgtaaggacagtttcaaagtgacatgaatggctactgcaaaaacgaagtaagttattctttctcagggcaaaattttgagtagattaattttgtttaaaaatgtgggacacagtcgtcaagtcttttgaactatctaagagatatgttgaaaagagaataattttattattaaatgagctatggaaagtccagcttttttctttaccttttttttatggtttcttctgttaagtgtaactggctattcagtttatgttagttttggtccaccttcaaaaaaattacgtgatccttttgaagaacacgaagattaaacaagttaaaaagtactatttttacaagtgacttcggtgcctctgagaaccctagttatagtgatataaaataactagctaactactttatatttttatgaaagtcattttgtcgagcatataaacaaaaacaaaattgctatactaggcagtcacagtgcaactgtctccgtctccttaaccgagaaagggtaaacgtcttcggtaaagtaacaaactttagttatgttaactgcttgcgagttaaccattttttttcctccgaaggacaacagttggcagttgccaaactttagtggtctaatatttatattaggcagttggcaggcaactgcactgacgtcccgaaggggaaggggtttacttacctcctaacggagtatataaatagaataaaatttatttcctgcgctagcagatttacatactaggattttaatactccgaaggaggcagtggcggtaccactgccactggcgtcctccttccccttcgggcaaatgcattttagtgccacttaagtttacttgcctaggcagttggcaggacgtcagtggcagtggtaccgcgactgcctatatttatatactcctaagtttacttgcctaggcagttggcaggcaactgccactgacgtccttccccttccccttcgggacgtccccttacgggaatataaatattagtggatatttatatactgcgatgtttacatactccgaaggaggagagctagcagttgcctgccaactgcctaatataaatattgggcaagtaaacttagaatgtttacatactccgaaggaggacgtcccttacgggaatataaatattagtggcagtggtaccgccactgcctccttcggagtattaaaatcctagtatataatataccgtaagggacgtcctccgacggtggcagtggcggtaccactgccaccggcgtcctaatatacatattgaagtatttaaacctgttagcgcacgctctaacgagtcagtaaacttcccttttggggcttctaggcagcgcataaattttctaggacgaacgtccactggcgtctcgtaaggagcagtgacaggccactaatgtccccttaatgggtaaataaatggctatcgtctatccatgaagagaccatatattccagtagcaccgttatgatcctcaaagggtaacaccatttgtatagtattatggtgaaatgcatccctttcagggtagatttatatcttacaG

**References**

Young RE, Purton S. Cytosine deaminase as a negative selectable marker for the microalgal chloroplast: a strategy for the isolation of nuclear mutations that affect chloroplast gene expression. Plant J 2014, 80:915-925.

Young RE, Purton S. Codon reassignment to facilitate genetic engineering and biocontainment in the chloroplast of *Chlamydomonas reinhardtii*. Plant Biotechnol J 2016, 14:1251-1260.
